# Homologous Basal Ganglia Network Models in Physiological and Parkinsonian Conditions

**DOI:** 10.3389/fncom.2017.00079

**Published:** 2017-08-22

**Authors:** Jyotika Bahuguna, Tom Tetzlaff, Arvind Kumar, Jeanette Hellgren Kotaleski, Abigail Morrison

**Affiliations:** ^1^Institute of Neuroscience and Medicine (INM-6), Institute for Advanced Simulation (IAS-6), JARA Brain Institute I, Jülich Research Center Jülich, Germany; ^2^Computational Science and Technology, School of Computer Science and Communication, KTH Royal Institute of Technology Stockholm, Sweden; ^3^Faculty of Biology, Bernstein Center Freiburg, University of Freiburg Freiburg, Germany; ^4^Institute for Cognitive Neurosciences, Ruhr University Bochum, Germany

**Keywords:** basal ganglia, network models, degeneracy, oscillations, Parkinson's disease

## Abstract

The classical model of basal ganglia has been refined in recent years with discoveries of subpopulations within a nucleus and previously unknown projections. One such discovery is the presence of subpopulations of arkypallidal and prototypical neurons in external globus pallidus, which was previously considered to be a primarily homogeneous nucleus. Developing a computational model of these multiple interconnected nuclei is challenging, because the strengths of the connections are largely unknown. We therefore use a genetic algorithm to search for the unknown connectivity parameters in a firing rate model. We apply a binary cost function derived from empirical firing rate and phase relationship data for the physiological and Parkinsonian conditions. Our approach generates ensembles of over 1,000 configurations, or homologies, for each condition, with broad distributions for many of the parameter values and overlap between the two conditions. However, the resulting effective weights of connections from or to prototypical and arkypallidal neurons are consistent with the experimental data. We investigate the significance of the weight variability by manipulating the parameters individually and cumulatively, and conclude that the correlation observed between the parameters is necessary for generating the dynamics of the two conditions. We then investigate the response of the networks to a transient cortical stimulus, and demonstrate that networks classified as physiological effectively suppress activity in the internal globus pallidus, and are not susceptible to oscillations, whereas parkinsonian networks show the opposite tendency. Thus, we conclude that the rates and phase relationships observed in the globus pallidus are predictive of experimentally observed higher level dynamical features of the physiological and parkinsonian basal ganglia, and that the multiplicity of solutions generated by our method may well be indicative of a natural diversity in basal ganglia networks. We propose that our approach of generating and analyzing an ensemble of multiple solutions to an underdetermined network model provides greater confidence in its predictions than those derived from a unique solution, and that projecting such homologous networks on a lower dimensional space of sensibly chosen dynamical features gives a better chance than a purely structural analysis at understanding complex pathologies such as Parkinson's disease.

## 1. Introduction

Our understanding of the circuitry of the basal ganglia has been much refined in recent years due to the discovery of distinct sub-populations of neurons within nuclei previously assumed to be homogeneous (Gertler et al., 2008; Taverna et al., 2008; Planert et al., 2010; Mallet et al., 2012; Mastro et al., 2014) and additional projections between nuclei previously thought to be unconnected (Nadjar et al., 2006; Calabresi et al., 2014; Saunders et al., 2015). Whereas ideally we would instantly be able to incorporate these new findings into well validated computational models of the basal ganglia and determine the dynamical role of these sub-populations and projections, in practice this is not at all simple. Any attempt to model the basal ganglia rapidly runs into difficulties due to lack of knowledge, particularly with respect to the strengths of connections between nuclei. Although empirical data has been gathered on the strengths of many of the connections in the basal ganglia circuit, such as lateral inhibition in striatum (Taverna et al., 2008; Planert et al., 2010), many others remain uncertain (e.g., afferent and efferent projections of the GPe-arkypallidal neurons).

Faced with this high degree of under-specification, modelers typically choose one of two alternatives: make simplifying assumptions on the unknown parameters, or strive for a unique or locally optimal solution by performing an extensive and computationally demanding parameter fit with respect to a cost function based on desired model dynamics. The former approach has the disadvantage that there will always be a question mark remaining over whether the simplifying assumptions underlying the specific choice of model parameters were valid. While the robustness of obtained results with respect to the parameter choice has been extensively studied in the context of single-cell (e.g., Achard and De Schutter, 2006) and small network models (e.g., Prinz et al., 2004), this has hardly been done for large-scale networks, specifically basal ganglia.

The latter approach has the disadvantages that defining criteria tightly enough to allow a unique solution to be located does not take variability of biology into account; for example a measurement of spiking activity in a given area can vary substantially between animals and labs. Further, one can generally assume that an alternative solution would have been found, if the cost function had been defined slightly differently, with no assurances that the solutions would have been close in parameter space. Therefore even when a solution has been generated by this method, it is still unclear what other points in parameter space might have been selected by equally well motivated cost functions, or whether they might even be more representative of the system to be modeled. The existence of alternative solutions may reflect either the lack of constraints from experimental observations or the variability that is prevalent in biological systems (Gutenkunst et al., 2007), and clearly demonstrates the problems of trying to converge on a single “best” solution for a high-dimensional and substantially under-specified system.

In this study, we propose an alternative approach for dealing with the multiple uncertainties in the basal ganglia circuit. We perform an extensive parameter search, but instead of striving for a unique solution, we embrace the uncertainty and use a genetic algorithm to generate a large ensemble of network configurations for both physiological and parkinsonian conditions. This allows us to investigate the common dynamics of the whole ensemble of configurations, thus permitting a higher degree of confidence in the results than the standard approach of generating and examining only one configuration for each condition.

Specifically, we develop a mean field model (Section 3.1) with a structure that incorporates the recent findings on the Globus pallidus externus (GPe), indicating that it is organized into two distinct subpopulations (Mallet et al., 2008, 2012; Abdi et al., 2015). One of the subpopulations projects upstream to striatum, expresses preproenkephalin and fires in phase with cortical slow wave and beta activity in parkinsonian conditions. These neurons are termed as arkypallidal neurons or GPe tonically active (TA) neurons. The other subpopulation of neurons mostly projects downstream in basal ganglia, expresses parvalbumin and fires anti-phase with cortical slow wave and beta activity in parkinsonian conditions. These neurons are termed as prototypical or GPe tonically inactive (TI) neurons. The existence of two types of GPe neurons with distinct projection patterns and dynamics suggests that they might be parts of different functional pathways.

As indicated above, not much is known about the effective connectivities within these subpopulations as well as between these subpopulations and rest of the basal ganglia. Whereas a search for connectivity strengths of these newly discovered GPe subpopulations that produce consistent activity has been carried out in the recent and thorough modeling study by Nevado-Holgado et al. (2014), this analysis was restricted to the GPe-STN subcircuit and considered striatal input as feedforward inhibition. Since at least one of the GPe subpopulation is known to project massively upstream to striatum, the striatum forms a rather strong recurrent loop with GPe. This recurrency is bound to affect the parameter search and hence is integral to understand the role of these sub-populations. We therefore model a larger set of the basal ganglia nuclei and include the reciprocal connections.

Our genetic algorithm (Section 3.2) is configured to accept all network configurations that reproduce the experimentally observed network activity for either a physiological or parkinsonian condition (Mallet et al., 2008; Abdi et al., 2015). This process reveals a large number of valid configurations for both conditions, with substantial variation in the values found for the free connectivity parameters (Section 3.3). We call these valid configurations *homologies* or *homologous networks*, since for different values of effective connectivities, they result in similar dynamics. We further investigate the significance of the homologies by replacing the individual parameter values by the mean of the corresponding distribution, or alternatively shuffling parameter values between the members of an ensemble, and determining how many networks retain their original physiological or parkinsonian classification. We find that the classification of the networks is more sensitive to some parameters than others, and that shuffling values within a distribution causes more networks to become invalid, i.e., no longer fulfill the dynamic criteria for a physiological or parkinsonian network, than replacing the distribution by its mean. Moreover, if the parameters are shuffled cumulatively, the proportion of networks retaining their original classification monotonically decreases for both physiological and parkinsonian networks. We also observe that the sensitivity of parkinsonian network dynamics to manipulations of a given parameter strongly depend on how correlated that parameter is with other free parameters.

The generation of ensembles of network configurations also enables some predictions to be made about effective connectivities within the basal ganglia. Some of them are consistent with the known data [e.g., strengthened (weakened) cortical connections to D2-MSNs (D1-MSNs) in Parkinson's disease] and some of them are novel and require experimental validation (e.g., reduced self inhibition of GPe-TI in Parkinson's disease). These predictions are discussed in more detail in the Section 4.2.

To gain insight into the essential properties of the network configurations classified as physiological and parkinsonan, we project them onto a 2-dimensional space defined by dynamical properties of the network, namely its ability to suppress activity in the globus pallidus internus (GPi) and the network's susceptibility to oscillations in response to a square wave stimulus (Section 3.4). Although each parameter exhibits overlap in the distributions generated for the physiological and parkinsonian ensembles, this functional classification reveals distinct separation between the two ensembles.

Our results show that these dynamical features serve as good predictors of the network classification, and indicate that the discovered homologies do not come about merely as a result of lack of constraints used for the parameter search, but reflect common non-trivial dynamic properties. We conclude that the generation of large ensembles of valid network configurations based on simple dynamical features (such as rate and phase) and investigating their collective behavior with respect to higher level features (such as oscillation) is a fruitful method for acquiring insight into under-specified neural circuits, and gives a better chance at understanding complex pathologies like Parkinson's disease, which involves alterations to multiple pathways in the basal ganglia.

## 2. Materials and methods

### 2.1. Mean field model of the basal ganglia

We used a Wilson-Cowan model of the mean activity of seven basal ganglia nuclei, namely, D1-MSN (D1-medium spiny neuron), D2-MSN, FSI (fast spiking interneuron), GPe-TA (globus pallidus externus—tonically active), GPe-TI (GPe-tonically inactive), STN (subthalamic nucleus) and GPi (globus pallidus internus). The dynamics of the system is given by

(1) $$\begin{array}{l} \tau \dot{Y}=\overset{\text{leak}}{\overset{︷}{-Y}}+\;S\;(\overset{\text{recurrent}+\text{ext}.\;\text{input}}{\overset{︷}{A\cdot Y+B\cdot \lambda_{\text{CTX}}}}) \end{array}$$

with the population-firing-rate vector

$$Y\left(t\right)={\begin{array}{l} {[\lambda }_{\text{D}1}\left(t\right),\lambda_{\text{D}2}\left(t\right),\lambda_{\text{FSI}}\left(t\right),\lambda_{\text{TA}}\left(t\right),\lambda_{\text{TI}}\left(t\right),\lambda_{\text{STN}}\left(t\right),\lambda_{\text{GPi}}\left(t)\right] \end{array}}^{T}$$

and the sigmoidal activation function

(2) $$\begin{array}{l} S\left(x,\theta ,\lambda_{\text{max}}\right)=\frac{\lambda_{\text{max}}}{1+e^{-a\left(x-\theta \right)}}. \end{array}$$

Here, λ_CTX_ denotes the firing rate of cortical inputs, $Ẏ=\frac{dY}{dt}={\left[\frac{d\lambda_{\text{D}1}}{dt},\frac{d\lambda_{\text{D}2}}{dt},\frac{d\lambda_{\text{FSI}}}{dt},\frac{d\lambda_{\text{TA}}}{dt},\frac{d\lambda_{\text{TI}}}{dt},\frac{d\lambda_{\text{STN}}}{dt},\frac{d\lambda_{\text{GPi}}}{dt}\right]}^{T}$ the temporal derivative of the rate vector *Y*(*t*), and ^*T*^ the matrix transpose. For the sake of simplicity, the time constant τ = 15 ms is kept constant for all nuclei. To test the sensitivity of the results to this choice, parameter search and simulations are also performed for τ = 1ms. This produces qualitatively similar results, but shifts the frequency of beta oscillations (peaks in spectra in **Figure 6D**) out of the experimentally observed range (data not shown).

The values of θ and λ_max_ (Table 1) were chosen in order to get realistic distributions of instantaneous firing rates under different input conditions (Supplementary Figure 2). For a specific nucleus, λ_max_ and θ are fixed across all network configurations and for both physiological and pathological conditions. Activity is modeled assuming zero delays (i.e., instantaneous update), as this is convenient for the overall ease of analysis of the system. Moreover, oscillations in a circuit can be purely delay driven. As we are interested in the role of effective connectivities on oscillations, a choice of zero delay removes the confounding factor.

**Table 1:** Nucleus specific parameters for the sigmoidal activation function.

**Nucleus name**	**θ**	**λ_max_**
D1-MSN	0.1	65 (Kiyatkin and Rebec, 1999)
D2-MSN	0.1	65 (Kiyatkin and Rebec, 1999)
FSI	0.1	80
GPe-TA	0.4	75
GPe-TI	0.4	125
STN	0.4	500 (Kita et al., 1983; Nakanishi et al., 1987)
GPi	0.1	250 (Nakanishi et al., 1987; Hashimoto et al., 2003)

The coupling matrices

(3) $$\begin{array}{l} A=\left[\begin{array}{ccccccc} J_{\text{D}1,\text{D}1} & J_{\text{D}1,\text{D}2} & J_{\text{D}1,\text{FSI}} & J_{\text{D}1,\text{TA}} & J_{\text{D}1,\text{T}1} & 0 & 0\\ J_{\text{D}2,\text{D}1} & J_{\text{D}2,\text{D}2} & J_{\text{D}2,\text{FSI}} & J_{\text{D}2,\text{TA}} & J_{\text{D}2,\text{T}1} & 0 & 0\\ 0 & 0 & 0 & J_{\text{FSI},\text{TA}} & J_{\text{FSI},\text{T}1} & 0 & 0\\ 0 & J_{\text{TA},\text{D}2} & 0 & J_{\text{TA},\text{TA}} & J_{\text{TA},\text{T}1} & J_{\text{TA},\text{STN}} & 0\\ 0 & J_{\text{T}1,\text{D}2} & 0 & J_{\text{T}1,\text{TA}} & J_{\text{T}1,\text{T}1} & J_{\text{T}1,\text{STN}} & 0\\ 0 & 0 & 0 & J_{\text{STN},\text{TA}} & J_{\text{STN},\text{T}1} & 0 & 0\\ J_{\text{GPi},\text{D}1} & 0 & 0 & 0 & J_{\text{GPi},\text{T}1} & J_{\text{GPi},\text{STN}} & 0 \end{array}\right] \end{array}$$

and

(4) $$\begin{array}{l} B={\left[\begin{array}{c} J_{\text{D}1,\text{CTX}},J_{\text{D}2,\text{CTX}},J_{\text{FSI},\text{CTX}},0,0,J_{\text{STN},\text{CTX}},0 \end{array}\right]}^{T} \end{array}$$

denote the recurrent and input connection matrices, respectively. An element *J*_i,j_ of *A* or *B* represents the effective strength of the connection from population *j* to population *i*. The colors of the parameters in Equations (3) and (4) indicate whether a particular projection is considered as a free (red) or fixed (blue) parameter. The calculation of the fixed parameters is described in detail in Section 2.1.1. The code for this model and analysis scripts are shared in the git repository https://github.com/jyotikab/Homology_BG.git.

#### 2.1.1. Fixed parameters

The effective connectivity parameters considered fixed in coupling matrix *A* (*J*_*i,j*_ in blue) are calculated from the experimental data used to tune the spiking network model presented in Lindahl et al. (2013). We did not perform a direct conversion from the parameters of the spiking model, since it is non-trivial to calculate the effective connectivities analytically from the adaptive exponential integrate and fire model used in the paper. The analytical method to calculate the effective connectivity assumes a LIF neuron, with membrane potential dynamics given by

(5) $$\begin{array}{l} \tau_{\text{m}}\overset{\cdot }{v}=-v+Ri\left(t\right) \end{array}$$

where τ_m_ is the membrane time constant and $R=\frac{\tau_{\text{m}}}{C}$ is the membrane resistance. The impulse response for a delta input (*Ri*(*t*) = δ(*t*)) can be calculated as :

(6) $$\begin{array}{l} h\left(t\right)=\frac{1}{\tau_{\text{m}}}e^{\frac{-t}{\tau_{\text{m}}}}\Theta \left(t\right) \end{array}$$

with Θ(*t*) = 1 for *t* ≥ 0 and 0 otherwise. A post synaptic current (PSC) depends on the synaptic conductance *G*(*t*), reversal potential of the synaptic connection (*E*_rev_) and potential at which the membrane is clamped, also known as the holding potential (*V*_hold_).

(7) $$\begin{array}{l} I=G\left(t\right)\left(E_{\text{rev}}-V_{\text{hold}}\right) \end{array}$$

Assuming exponential currents with a decay constant τ_s_

(8) $$\begin{array}{l} i\left(t\right)=Ie^{\frac{-t}{\tau_{\text{s}}}}\Theta \left(t\right) \end{array}$$

then the postsynaptic potential can then be calculated as the convolution of neuron's impulse response and the incoming PSC

(9) $$\begin{array}{l} v\left(t\right)=\left(i*h\right)\left(t\right)=RI\frac{\tau_{\text{s}}}{\tau_{\text{s}}-\tau_{\text{m}}}\left(e^{\frac{-t}{\tau_{\text{s}}}}-e^{\frac{-t}{\tau_{\text{m}}}}\right)\Theta \left(t\right) \end{array}$$

The effective connectivity per synapse is the total area of the PSP, which can be calculated by integrating

(10) $$\begin{array}{l} \bar{v}=\int_{0}^{\infty }dtv\left(t\right) \end{array}$$

In order to calculate the total effective connectivity, the integrated PSP needs to be multiplied by the number of synapses or in-degree (*K*) of the circuit.

(11) $$\begin{array}{l} J=K\bar{v} \end{array}$$

Some experimental studies report the average integrated PSPs of a synapse, in which case the effective connectivity is calculated by simply scaling it with the in-degree as above.

However, often connectivity strengths are reported in other units. Some studies report just the average amplitude of the PSP (*V*_psp_), so an integrated PSP is calculated by multiplying the PSP with synaptic time constant (assuming an exponential shape PSP) i.e.,

(12) $$\begin{array}{l} J=KV_{\text{psp}}\tau_{\text{s}} \end{array}$$

Other studies measure post synaptic currents (*I*_psc_) or integrated PSCs, i.e., the total charge (*Q* = *I*_psc_τ_s_) in which case, we calculate the effective connectivity by multiplying the integrated PSCs with the input resistance *R* and in-degree, i.e.,

(13) $$\begin{array}{l} J=KRI_{\text{psc}}\tau_{\text{s}} \end{array}$$

(14) $$\begin{array}{l} =KRQ \end{array}$$

Finally, if the experiment measures conductance instead of PSCs, the effective connectivity can be estimated by calculating the PSC for a given holding potential (*V*_hold_) and reversal potential of the synaptic connection (*E*_rev_) as *I*_psc_ = *G*(*t*)(*E*_rev_ − *V*_hold_) τ_s_, leading to

(15) $$\begin{array}{l} J=KRG\left(t\right)\left(E_{\text{rev}}-V_{\text{hold}}\right)\tau_{\text{s}} \end{array}$$

##### Estimation of *J*_GPi,D1_

The data for D1-MSNs connections to GPi were estimated from Chuhma et al. (2011). This study measures the functional connectivity between MSNs and their afferent and efferent nuclei in brain slices of bidirectional tetO-rhodopsin (BTR) mice. This is done by optogenetically stimulating either the afferent nuclei or incoming fibers from the afferent nuclei while recording IPSCs from the target nucleus. The effective connectivity is hence calculated from Equation (13) (row 1 in Table 2). This value does not need to be scaled by an in-degree since the method aimed to stimulate all incoming projections to a target cell; hence the recorded post-synaptic current is indeed the effective connectivity as perceived by the cell of the target nucleus. The value however, had to be scaled up by ten, as suggested by Chuhma et al. (2011) since only 10% of MSNs expressed ChR2 in BTR mice. This is indicated by a multiplier of “×10” for the IPSCs value in row 1 in Table 2.

**Table 2:** Calculation of the fixed parameters.

**No**.	**Conn**.	***Q*_*ij*_ (pA.ms)**	***K*_*ij*_**	***I*_psc_ (pA)**	***R*_*i*_ (MΩ)**	**G (nS)**	***V*_hold_ (mV)**	***E*_rev_**	***V*_psp_ (mV)**	**τ_*ij*_ (ms)**	**$\bar{v}$ (mVs)**	***J*_*ij*_ (mVs)**
1	*J*_GPi,D1_	−	−	276.3 × 10 [1]	141.7 [1]	−	−	−	−	7.1 [2]	−	−2.8
1a	*J*_GPi,D1_	−	25^*^	−	144 [6]	3 × 2 [6]	−60 [6]	−80 [6]	−	5.2[6]	−	−2.2
2	*J*_GPi,STN_	−	106[9]	−	97.3[5]	−	−	−	1.4 [5]	1.6 [5]	−	0.24
2a	*J*_GPi,STN_	900[4]	3^*^	−	97.3[5]	−	−	−	−	4.2 [4]	−	0.26
3	*J*_GPi,GPe_	−	32 [3]	−	144 [6]	20 × 0.2	−60 [6]	−72 [6]	−	2.1[6]	−	−0.78
4	*J*_D1,D1_	−	208.25^*^[9]	−	−	−	−	−	0.24 [7]	14 [8]	−	−0.69
4a	*J*_D1,D1_	−	291.8^*^[9]	−	−	−	−	−	−	−	0.055 [8]	−16.
4b	*J*_D1,D1_	−	7.3^*^[12]	−	−	−	−	−	−	−	0.055 [8]	−0.4
5	*J*_D1,D2_	−	386.75^*^[9]	−	−	−	−	−	0.27 [7]	11 [8]	−	−1.15
5a	*J*_D1,D2_	−	303.1^*^[9]	−	−	−	−	−	−	−	0.223 [8]	−67.6
5b	*J*_D1,D2_	−	7.6^*^[12]	−	−	−	−	−	−	−	0.223 [8]	−1.7
6	*J*_D2,D2_	−	497.6^*^[9]	−	−	−	−	−	0.45 [7]	13 [8]	−	−2.9
6a	*J*_D2,D2_	−	510^*^[9]	−	−	−	−	−	−	−	0.117 [8]	−59.67
6b	*J*_D2,D2_	−	12.8^*^[12]	−	−	−	−	−	−	−	0.117 [8]	−1.5
7	*J*_D2,D1_	−	97.3^*^[9]	−	−	−	−	−	0.33 [7]	10 [8]	−	−0.32
7a	*J*_D2,D1_	−	85^*^[9]	−	−	−	−	−	−	−	0.078 [8]	−6.63
7b	*J*_D2,D1_	−	2.14^*^[12]	−	−	−	−	−	−	−	0.078 [8]	−0.16
8a	*J*_MSN-MSN_	−	−	103.8 × 10 [1]	231 [1]	−	−	−	−	13 [8]	−	−3.35
8b	*J*_MSN-MSN_	−	595[9]	−	−	−	−	−	0.45 [7]	13 [8]	−	−3.48
9	*J*_D1,FSI_	−	27 [10]	−	−	−	−	−	4.5 × 0.6 [7]	8.1 [11]	−	−0.65
9a	*J*_D1,FSI_	−	27 [10]	501 × 0.6 [11]	142 [11]	−	−	−	−	8.1 [11]	−	−0.06
10	*J*_D2,FSI_	−	20.3^*^[10]	−	−	−	−	−	3.1 × 0.6 [7]	7.6 [11]	−	−0.3
10a	*J*_D2,FSI_	−	18.3^*^[10]	578 × 0.6 [11]	142 [11]	−	−	−	−	7.6 [11]	−	−0.04

*The non-grayed rows are the values used in the model. The grayed rows are alternative methods for estimating the connection strengths using the parameters from other studies. Numbers marked with an asterisk had to be calculated, see main text for details. Data sources: [1], Chuhma et al. (2011); [2], Borgkvist et al. (2015); [3], Lindahl et al. (2013); [4], Ammari et al. (2010); [5], Nakanishi et al. (1997); [6], Connelly et al. (2010); [7], Planert et al. (2010); [8], Taverna et al. (2008); [9], Steiner and Tseng (2017); [10], Koós and Tepper (1999); [11], Gittis et al. (2010); [12], López-Huerta et al. (2013)*.

The value of synaptic time constant τ_GPi,D1_ was obtained from Borgkvist et al. (2015). Connelly et al. (2010) also measured the strength of striatonigral projection, but in paired recordings and in form of synaptic conductance. Since the striatonigral connections undergo short term facilitation, the conductance is scaled by a factor of two, as indicated by a “×2” in row 1a. If we assume an in-degree of 500 as estimated by Lindahl et al. (2013), the effective connectivity calculated using the data from Connelly et al. (2010) gives much higher values. Since Connelly et al. (2010) specifically mentioned that they were unable to measure clear unitary striatonigral connections during minimal striatal stimulation, the measured synaptic response might represent a combined effect of multiple incoming projections. With an in-degree of 25, the effective connectivity calculated is in the same range as the value from Chuhma et al. (2011) (grayed row 1a in Table 2).

##### Estimation of *J*_GPi,STN_

The effective connectivity for STN projections to GPi were estimated from Nakanishi et al. (1997), who measured EPSCs in SNr during STN stimulation. An in-degree of 106 synapses was used as estimated in Steiner and Tseng (2017) and effective connectivity is calculated using Equation (12) (row 2 in Table 2). In order to further verify this value, the effective connectivity was calculated from Ammari et al. (2010), who measured synaptic strength in terms of total charge (*Q*_GPi,STN_) in a completely preserved basal ganglia slice (BGS). Since this study did not measure the input resistance of GPi, the value from Nakanishi et al. (1997) was used. The effective connectivity is calculated using Equation (14) (row 2 in Table 2). However, with an in-degree of 106, the values are around 50 times larger than values calculated from Nakanishi et al. (1997). However, since Ammari et al. (2010) used a basal ganglia slice and STN was stimulated using bipolar electrodes, the synaptic strength calculated cannot be interpreted as if measured in a paired recording. If the in-degree is rescaled to 3, a value is obtained in the range of Nakanishi et al. (1997) (row 2a in Table 2).

##### Estimation of *J*_GPi,GPe_

The aforementioned study by Connelly et al. (2010) also measures pallidonigral connections (*J*_GPi,GPe_) in paired recordings in terms of synaptic conductance. We used this data to calculate the effective connectivity of pallidonigral connections in a similar method to striatonigral effective connectivity (Equation 15 and row 3 in Table 2). Since this connection undergoes short term depression, it is rescaled by 0.2, where the PSP reaches a steady state after the initial short term plasticity. This is indicated as “× 0.2” in the conductance.

##### Estimation of lateral inhibition in striatum (*J*_D1,D1_, *J*_D1,D2_, *J*_D2,D2_, *J*_D2,D1_)

The striatal MSNs are classified into two groups depending on the dopamine receptor they express, i.e., D1-MSNs and D2-MSNs. Data on the synaptic strengths and connectivity is available separately for these category pairs as well as unseparated MSN pairs. The effective connectivity between D1 and D2-MSNs was calculated using data from Planert et al. (2010). The in-degree was calculated by rescaling the in-degree for unclassified MSN (*K*_MSN_ = 595 synapses per MSN cell—Steiner and Tseng, 2017) by the connectvity of classified MSN pairs, e.g.,

(16) $$\begin{array}{l} K_{\text{D}1,\text{D}1}=\frac{\rho_{\text{D}1,\text{D}1}}{\rho_{\text{D}1,\text{D}1}+\rho_{\text{D}1,\text{D}2}}K_{\text{MSN}} \end{array}$$

For values of ρ_D1,D1_ and ρ_D1,D2_ of 0.07 and 0.13 resp. (from Planert et al., 2010), the scaled indegree (*K*_D1,D1_) for D1-D1-MSNs is 208.25. The effective connectivity of all the four connections was calculated using Equation (12) (rows 4–7 in Table 2).

We also estimated effective connectivity for an average MSN-MSN connection from data supplied by Chuhma et al. (2011), which after scaling up by ten, gives a value of approximately −3.4 (row 8a). This value is strongly supported by effective connectivity calculated for MSN-MSN PSPs from Planert et al. (2010), resulting in approximately −3.5 (row 8b). Due to the paucity of projections from D1-MSNs and their weak synaptic strengths, it is likely that on an average a measured MSN-MSN connection is a projection from D2-MSN (either to D2-MSN or D1-MSN). This corresponds to a mean effective connectivity of −2.0 (refer to *J*_D2,D2_, *J*_D1,D2_, with means of −2.9 and −1.15). This fits well with the value estimated from Chuhma et al. (2011) and Planert et al. (2010).

The work by Taverna et al. (2008) measures the integrated PSPs ($\bar{v}$) between D1-D2MSNs, but when these values are multiplied by the in-degree, the values obtained are ten times larger than the values calculated above (row 4a, 5a, 6a, 7a). A possible explanation for this is that the analysis in Taverna et al. (2008) is limited to IPSCs with a short rise time (<1ms) that likely arise from proximal synapses. The distal synapses, as suggested by Taverna et al. (2008), might show different properties. If they were weaker, they would reduce the average measured integrated PSP for the neuron pairs, thereby yielding an effective connectivity in the correct range. Alternatively, we used the distance dependent connectivity data measured by López-Huerta et al. (2013), that suggests that in a slice of around 100μm from surface, as also used by Taverna et al. (2008), the in-degree drops to around 5–15 per MSNs. Using *K*_MSN_ = 15 in Equation (16), the in-degree for D1-D1MSNs (*K*_D1,D1_) is 7.3. Recalculating the effective strengths with this in-degree, we get a good match to the values obtained by Planert et al. (2010) (rows 4b, 5b, 6b, 7b).

##### Estimation of interneuronal inhibition to striatum (*J*_D1,FSI_, *J*_D2,FSI_)

The fast spiking interneurons (FSIs), in spite of being only 2% in number, provide strong feedforward inhibition to the striatal MSNs. We estimated their effective inhibition to D1 and D2-MSNs using the data provided by Planert et al. (2010). The in-degree was calculated by scaling the average in-degree of unclassified MSNs, which is 4-27 FSI neurons per MSN (Koós and Tepper, 1999). Since Planert et al. (2010) report that FSIs preferentially inhibit D1-MSNs, we assume that the in-degree to D1-MSNs (*K*_D1,FSI_) is 27, with the in-degree to D2-MSNs being scaled accordingly

(17) $$\begin{array}{l} K_{\text{D}2,\text{FSI}}=\frac{\rho_{\text{D}2,\text{FSI}}}{\rho_{\text{D}1,\text{FSI}}}K_{\text{MSN-FSI}} \end{array}$$

With the values of ρ_D2,FSI_ = 0.89, ρ_D1,FSI_ = 0.67 and *K*_MSN-FSI_ = 27, the rescaled of *K*_D2,FSI_ is estimated as 20.3.

The short term depression in FSI synapses to MSN is indicated by a factor of “×0.6” in the *V*_psp_ column. The calculated effective connectivities are listed in rows 9 and 10 of Table 2. The synaptic time constants were taken from an another study (Gittis et al., 2010), that concurs with the finding of Planert et al. (2010) that FSIs inhibit D1-MSNs more than D2-MSNs. They measure the synaptic strengths in terms of post-synaptic currents, which are used to calculate the effective connectivities as shown in the table (rows 9a and 10a). Although these values preserve the qualitative relationship of effective inhibition to D1 and D2-MSNs (i.e., *J*_D1,FSI_ > *J*_D2,FSI_), they are ten times smaller than those estimated from the data of Planert et al. (2010) (rows 9a and 10a). This maybe because of large variance in inhibition from FSIs to MSNs as observed by both studies [Planert et al. (2010): 4.8 mV±4.9, 3.1 mV±4.1 ; Gittis et al. (2010): 501pA±760, 578pA±834]. We chose to use the values from Planert et al. (2010), because we use the values from this study to estimate the other four striatal corrections (see Section 2.1.1).

### 2.2. Effective connectivity parameter search

We used a genetic algorithm to find valid network configurations that reproduce experimental observed phase and activity relationships in physiological and Parkinsonian conditions. The pseudocode is given in Algorithm 1.

**Algorithm 1:**
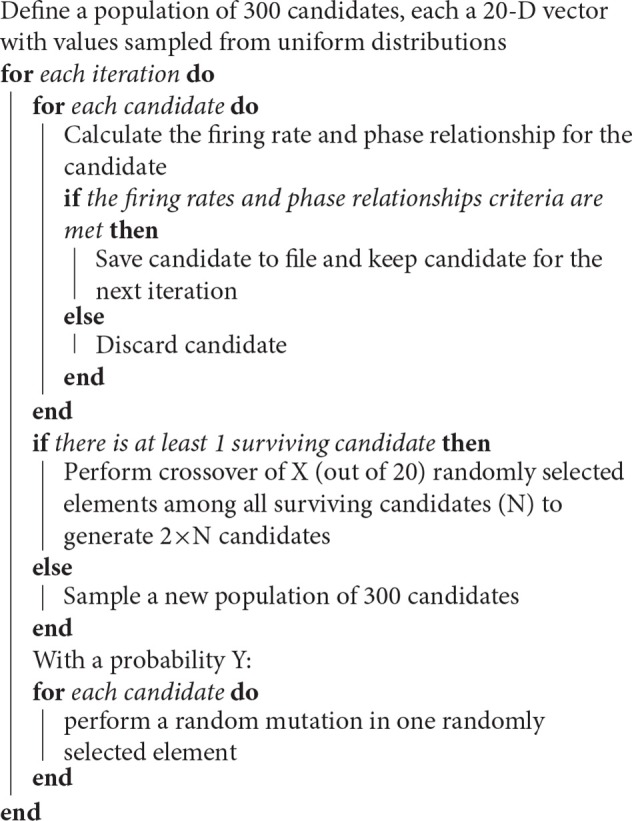
Pseudocode of the genetic algorithm generating network configurations.

The crossover number *X* (number of elements among the 20 free parameters to be swapped) and the random mutation probability *Y* (probability of a random mutation) were tuned in order to cover a reasonable amount of parameter space in a time span of 3 days (≈3,000 iterations). A value of *X* = 2 and *Y* = 0.1 gave the best results (data not shown). The range for all the negative parameters was (0, −6.) and positive parameters was (0, 13). In the case of a random mutation, the new value was drawn from the corresponding positive or negative uniform distribution. The parameter search was run for three different seeds to ensure that the results didn't depend on the initial conditions. The distributions are shown in Supplementary Figure 1.

The cost function used in the parameter search is binary rather than real-valued. We derive a series of phase and activity relationships based on experimental findings on the response of the GPe-nuclei (arkypallidal and prototypical) to cortical slow wave activity (SWA) and beta activation (Beta) (Mallet et al., 2008; Abdi et al., 2015). The SWA was recorded under the influence of anesthesia, whereas the cortical activity showed beta activation during hindpaw stimulation of the animal. For each candidate network configuration, we set λ_CTX_ in Equation (1) to provide SWA and beta stimulus as defined in Table 3. We then test whether the network's activity fulfills the relationships set out in Table 4. If it fulfills all the criteria in the left column, the network configuration is considered a valid example of a physiological network. If it fulfills all the criteria in the right column, it is considered a valid example of a parkinsonian network. If neither, than the configuration is discarded (see Algorithm 1).

**Table 3:** Cortical input parameters.

**Input type**	**Frequency (Hz)**	**Amplitude**
SWA (Slow wave activity)	2	2.0 (Ballion et al., 2008; Nevado-Holgado et al., 2014)
Beta (Beta Activation)	20	2.5 (Mallet et al., 2005; Nevado-Holgado et al., 2014)

**Table 4:** Criteria for classifying physiological and parkinsonian activities as shown in Mallet et al. (2008) and Abdi et al. (2015).

**No**.	**Property**	**Physiological**	**Parkinsonian**
(1)	λ_TI(SWA)_	[9.5, 45] spikes/s	[19, 35] spikes/s
(2)	λ_TI(Beta)_	[12,50] spikes/s	[7,19] spikes/s
(3)	λ_TA(Beta)_	[5,25] spikes/s	[7,15] spikes/s
(4)	λ_TA(SWA)_	[0,5] spikes/s	[1,6] spikes/s
(5)	λ_GPe_=λ_TA_+λ_TI_	λ_GPe(SWA)_ < λ_GPe(Beta)_	λ_GPe(SWA)_ > λ_GPe(Beta)_
(6)	Corr(λ_STN(SWA)_,λ_CTX(SWA)_)	>0	>0
(7)	FF(λ_TA(SWA)_)	<1	>1
(8)	Corr(λ_TA(SWA)_,λ_STN(SWA)_)	−	>0
(9)	FF(λ_TI(SWA)_)	<1	>1
(10)	Corr(λ_TI(SWA)_,λ_STN(SWA)_)	−	<0

*Square brackets represent ranges of firing rates*.

The (Pearson's) cross-correlation coefficients Corr(λ_x_,λ_y_) in Table 4 are used to test phase relationships between the activities of the nuclei x and y. A positive cross-correlation between the activities of STN (λ_STN_) and GPe-TA (λ_TA_), for example, imply an in-phase relationship. Although cross-correlation is not a direct test for phase relationship, it is sufficient in this case since the input is sinusoidal. The phase differences between λ_TA/TI_ and λ_STN_ in **Figures 2C,D** are calculated from the Fourier transformed signals (using Fast Fourier Transform of the NumPy library) at stimulus frequency. For physiological networks, the experimental data suggests that GPe-TA and GPe-TI are largely non-modulated by the cortical activity (Mallet et al., 2008). This was imposed by selecting the networks with low Fano factor

(18) $$\begin{array}{l} \text{FF}=\frac{\text{Var}\left(\lambda_{\text{TA/TI}}\right)}{\text{Mean}\left(\lambda_{\text{TA/TI}}\right)} \end{array}$$

for the GPe-TA and GPe-TI activity. Here, Var(·) and Mean(·) correspond to the variance across time and the time average, respectively. For parkinsonian conditions, the experimental data shows that λ_TA_ and λ_TI_ are strongly modulated by cortical activity which is reflected in a significant (positive/negative) cross-correlation and large Fano factors.

### 2.3. GPi suppression and susceptibility to oscillations

GPi suppression (GS) measures the ability of the network dynamics to effectively suppress the GPi activity and is defined as the ratio of the change in average GPi activity after the stimulus onset t^*^ to the activity before stimulus onset, i.e.,

(19) $$\begin{array}{l} \text{GS}=\frac{{\langle \lambda_{\text{GPi}}\left(t\right)\rangle }_{\left[t^{*}-\Delta_{1},t^{*}\right]}-{\langle \lambda_{\text{GPi}}\left(t\right)\rangle }_{\left[t^{*},t^{*}+\Delta_{2}\right]}}{{\langle \lambda_{\text{GPi}}\left(t\right)\rangle }_{\left[t^{*}-\Delta_{1},t^{*}\right]}} \end{array}$$

where Δ_1_ = Δ_2_ = 500 ms.

Susceptibility to oscillations (SO) denotes the tendency of the system to oscillate under a transient stimulus (here a square wave). It is calculated as one minus the mean spectral energy, i.e.,

(20) $$\begin{array}{l} \text{SO}=1-\text{SE} \end{array}$$

(21) $$\begin{array}{l} \text{SE}={\langle {\text{SE}}_{i}\rangle }_{i}\; \end{array}$$

(22) $$\begin{array}{l} \;{\text{SE}}_{i}=\frac{-\sum_{\omega }|\Lambda_{i}\left(\omega \right)|\cdot \log_{e}|\Lambda_{i}\left(\omega \right)|}{\log_{e}N\left(\omega \right)} \end{array}$$

(23) $$\begin{array}{l} \Lambda_{i}\left(\omega \right)=𝔉\left(\lambda_{i}\left(t\right)\right)\left(\omega \right) \end{array}$$

where *i* ∈ {GPi, GPe-TA, STN, GPe-TI} and SE_*i*_ denotes the spectral entropy for the amplitude spectrum Λ_*i*_(ω) of nucleus *i*, calculated as the Fourier transform of the activity, λ_*i*_.

We measure these dynamical properties of the system following a perturbation with a single square pulse of amplitude 4 spikes/s. Analogous results were obtained for amplitudes in the range 1–8 spikes/s (data not shown). Note that the square pulse input contains components in all frequency bands and is therefore an appropriate test stimulus for susceptibility to oscillations.

### 2.4. Manipulations of parameter distributions

The distributions of values generated for the free effective connectivity parameters are manipulated in two ways, “replace by mean” and “shuffle.” The manipulations are carried out either individually (one parameter at a time, restoring the original values of a parameter after each manipulation) or cumulatively (manipulating each parameter in succession, without restoring the original values of previously considered parameters).

#### 2.4.1. Replace by mean

For a given connection *J*_xy_, the specific value of that connection in all network configurations is replaced by the mean of that connection taken across all network configurations in that ensemble, i.e.,

$$J_{\text{xy}}=\frac{1}{M}\overset{M}{\underset{i=1}{\sum }}J_{\text{xy}}^{i}$$

where *M* = 1, 214 for the physiological ensemble and 1, 265 for the parkinsonian ensemble.

#### 2.4.2. Shuffle

For a given connection *J*_xy_, the specific values of that connection are randomly permutated between all members of the corresponding ensemble. This procedure was repeated for ten independent permutations.

### 2.5. Simulation and data analysis tools

All network simulations and the genetic algorithm are implemented in Python (http://www.python.org). Results were analyzed using SciPy and NumPy libraries and visualizations were carried out using Matplotlib (Hunter, 2007).

## 3. Results

### 3.1. Firing rate model of the basal ganglia

**Figure 1:**
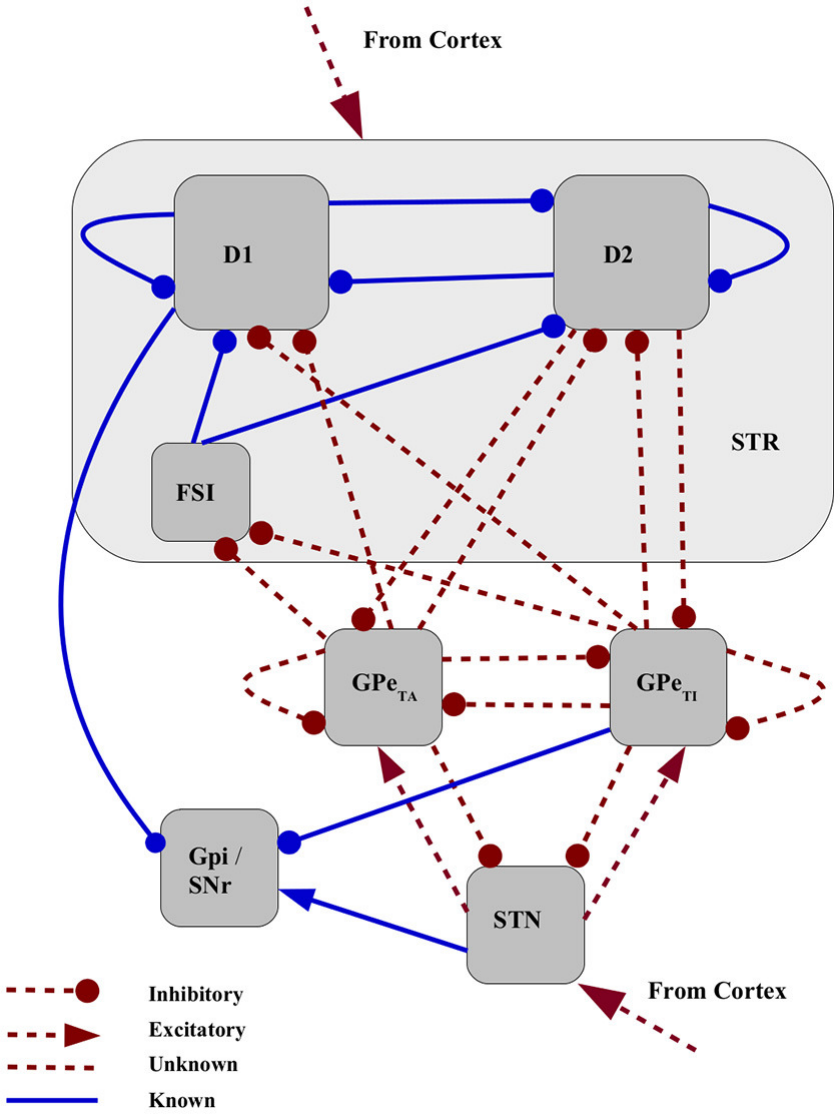
Network schematic for mean field model of basal ganglia. Connectivity strengths that can be estimated from experimental data are are fixed in this study shown as blue solid lines. The strengths of red dashed projections are considered to be free parameters in the model.

We developed a firing rate model of the basal ganglia consisting of seven neuronal populations, namely, D1-MSN(D1-medium spiny neuron), D2-MSN, FSI (fast spiking interneuron), GPe-TA (globus pallidus externus - tonically active, or arkypallidal), GPe-TI (GPe-tonically inactive, or prototypical), STN (subthalamic nucleus) and GPi (globus pallidus internus) as shown in Figure 1. These nuclei were chosen with the purpose of modeling a minimal basal ganglia circuit implementing the three main functional pathways, i.e., the direct pathway (D1 → GPi), the indirect pathway (D2 → GPe → GPi) and the hyperdirect pathway (STN → GPi). For the sake of simplicity, we limit our analysis to the basal ganglia dynamics and its output. Consequently, other connected nuclei such as thalamus are not represented, and cortex is modeled as a feedforward excitatory input. Consistent with the recent experimental findings (Mallet et al., 2012; Abdi et al., 2015) we modeled GPe as a network of two subpopulations. These subpopulations are distinct in terms of their response to cortical activity and may be part of different functional pathways. Since the strengths of the majority of the projections emanating from and projecting to GPe-TA and GPe-TI are unknown, they are considered to be free parameters (marked as red dashed lines in Figure 1). These include projections to striatum (D2-MSNs, D1-MSNs and FSIs), projections from D2-MSNs to GPe and recurrent projections between GPe and STN. Although it has been suggested that GPe-TA populations (arkypallidal) projects upstream to striatum much more than GPe-TI population (prototypical), here both are included as free parameters in order to include the possibility of few but strong projections from prototypical neurons to striatum. Similarly, the projections from both arkypallidal and prototypical neurons to STN are considered, even though it is assumed that the prototypical population projects downstream. All the cortical projections, i.e., to the striatum as well as to STN are also considered as free parameters, since their relative effective strengths are also unknown. The model dynamics and structure, and the estimation of the fixed connectivity parameters (blue solid lines in Figure 1), are defined in Section 2.1 in Materials and Methods.

### 3.2. An ensemble of network model configurations

The firing rate model described in Sections 2.1 and 3.1 and is incompletely specified due to the large number of unknown connectivity strengths. To address this issue, rather than making assumptions to specify these, we generate a large ensemble of model configurations by performing a parameter search across the 20 free parameters (see matrices *A* and *B* in Section 2.1). A genetic algorithm is used to find configurations of the free parameters shown in Figure 1 that conform to a series of criteria based on their activity and phase relationships (see Table 4 in Materials and Methods). A configuration is considered valid if the firing activity and phase relationships of arkypallidal and prototypical GPe subpopulations fit the experimental observations made for healthy and 6OHDA-lesioned rats (i.e., modeling Parkinson's disease) presented in Abdi et al. (2015) and Mallet et al. (2008).

**Figure 2:**
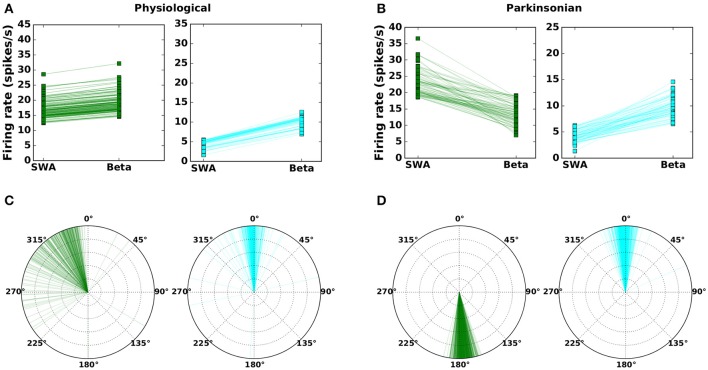
Mean firing rates **(A,B)** and phase difference with respect to STN activity **(C,D)** for prototypical neurons (GPe-TI; green) and arkypallidal neurons (GPe-TA; cyan) during slow-wave (SWA) and beta activity (Beta). Left column: physiological condition. Right column: parkinsonian condition. Each line (symbol) corresponds to one network configuration resulting from the genetic algorithm. For visual clarity, only 25% of network configurations are shown. Compare results to Figures 6–10 in Abdi et al. (2015).

The method is described in Section 2.2 and resulted in 1,214 network configurations classified as “physiological” and 1,265 classified as “parkinsonian.” The corresponding activity and phase relationships are shown in Figure 2 (c.f. Figures 6 and 10 in Abdi et al., 2015). In the physiological condition, the beta activation in cortex tends to increase the average activity in both arkypallidal and prototypical subpopulations (Figure 2A) and both populations mostly fire in-phase with the STN and cortical activity (Figure 2B). In the parkinsonian condition, however, beta activation has an opposite effect on the subpopulations. The activity in prototypical neurons decreases, whereas the activity in arkypallidal neurons increases, when the cortical activity switches from SWA (slow wave activity) to beta activation (Figure 2C). The subpopulations also differ in their phase relationship with STN activity in the parkinsonian condition, with arkypallidal neurons firing in-phase and prototypical neurons firing anti-phase with STN activity (Figure 2D).

It should be noted that every data point in Figures 2A,C indicates the activity of the arkypallidal and prototypical populations in one example network (i.e., one valid parameter configuration that fits the criteria). In contrast, each data point shown in Figures 6 and 10 in Abdi et al. (2015) corresponds to a different recorded neuron. Since this experimental data was gathered from many animals (20 healthy and 16 6OHDA-lesioned), these neurons may well belong to different homologous networks. It is also noteworthy that the model results are consistent with experimental results even in an aspect not used as a validity criterion (Table 4), namely that the phase differences in physiological networks show a wider distribution (with a peak ≈ 0°, but some points showing a phase difference of 270° or 180°), as compared to the narrow distribution of phase differences in parkinsonian networks (with peak ≈ 180° for prototypical and peak ≈ 0° for arkypallidal neurons). This might be due to relatively looser constraints on the phase difference in the heathy condition, where only weak modulation of arkypallidal and prototypical neurons by cortical activity is required (see Section 2.2).

**Figure 3:**
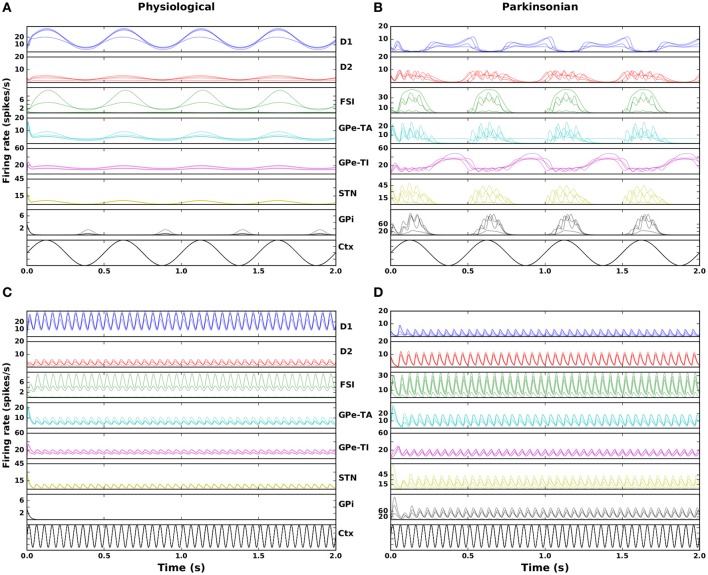
Nucleus specific response to cortical stimulation. Left column: physiological condition, right column: parkinsonian condition. **(A,B)** Activity in the seven modeled nuclei in response to cortical slow wave activity (black curve) for four randomly selected networks. **(C,D)** As in **(A,B)** but for cortical beta activity.

Figure 3 shows the response of the seven neuronal populations in the model to slow wave and beta activity in the cortex. The activity is shown for four randomly selected networks from both the physiological and parkinsonian classes. In the physiological condition (Figures 3A,C), both GPe-TA and GPe-TI show less modulation by cortical activity than in parkinsonian networks (Figures 3B,D), in which the opposite phase relationships between STN-arkypallidal (GPe-TA) and STN-prototypical (GPe-TI) can clearly be seen. Moreover, the parkinsonian networks exhibit an increase in GPi activity when cortical activity is high, whereas GPi mostly shows a decrease in its activity during high cortical activity in physiological networks, suggesting an anti-phase relationship between GPi and cortex (CTX). The parkinsonian networks also show a high frequency oscillation over the imposed slow wave activity unlike the physiological networks (Figures 3A,B), indicating an overall higher susceptibility to oscillations.

Table 5 gives the medians and quartiles of the effective connectivities generated for the physiological and parkinsonian ensembles. Although relationships between effective connectivities did not form part of the classification criteria, there is a good fit between many of the structural relationships observed in the generated ensembles and experimentally observed data. Some of the matches to well explored structural relationships include:

- Corticostriatal projections to D1-MSNs are stronger than those to D2-MSNs in physiological networks, but the situation reverses in parkinsonian networks. This is in agreement with the general trend for Parkinson's disease, i.e., the indirect pathway becomes stronger than the direct pathway due to dopamine depletion (Flores-Barrera, 2010; Escande et al., 2016)
  (physiological: *J*_D1,CTX_ = 7.8, *J*_D2,CTX_ = 2.6; parkinsonian: *J*_D1,CTX_ = 2.7, *J*_D2,CTX_ = 9.7)
- Corticosubthalmic projections are weaker in parkinsonian networks. Although the hyperdirect pathway is known to strengthen in Parkinson's disease, corticosubthalamic projections are shown to break down, characterized by loss of vGluT1-positive terminals in STN of parkinsonian monkeys (Villalba et al., 2015) as also shown by our results. In rodent models, although there is no direct evidence for this prediction, it is corroborated by the observation that optogenetic stimulation of corticosubthalamic projections ameliorate bradykinesia and akinesia in 6-OHDA lesioned mice (Sanders and Jaeger, 2016)
  (physiological: *J*_STN,CTX_ = 3.8, parkinsonian: *J*_STN,CTX_ = 0.83)

**Table 5:** Medians and quartiles (25%, 75%) of the effective weight distributions for physiological and parkinsonian networks (cf. Figure 4A).

**Connection**	**Physiological**	**Parkinsonian**
*J*_D1,TA_	−0.83 (−0.18, −1.5)	−0.93 (−0.08, −2.0)
*J*_D1,TI_	−0.3 (−0.0, −0.79)	−0.18 (−0.0, −0.61)
*J*_D2,TA_	−1.2 (−0.4, −2.1)	−1.4 (−0.61, −2.3)
*J*_D2,TI_	−0.2 (−0.0, −0.76)	−0.6 (−0.16, −1.1)
*J*_FSI,TA_	−0.23 (−0.0, −0.8)	−0.23 (−0.0, −0.81)
*J*_FSI,TI_	−0.82 (−0.0, −1.75)	−1.0 (−0.04, −2.1)
*J*_TA,D2_	−0.4 (−0.0, −1.0)	−2.1 (−1.2, −4.3)
*J*_TI,D2_	−0.45 (−0.0, −1.0)	−1.6 (−1.2, −2.62)
*J*_TA,TA_	−0.6 (−0.0, −1.65)	−1.2 (−0.17, −2.2)
*J*_TA,TI_	−0.9 (−0.22, −1.6)	−0.5 (−0.0, −1.3)
*J*_TI,TA_	−0.27 (−0.0, −0.8)	−0.25 (−0.0, −0.71)
*J*_TI,TI_	−0.64 (−0.15, −1.2)	−0.03 (−0.0, −0.14)
*J*_STN,TA_	−0.75 (−0.0, −1.41)	−0.4 (−0.0, −0.81)
*J*_STN,TI_	−2.0 (−1.2, −2.8)	−1.2 (−0.54, −1.9)
*J*_TI,STN_	0.92 (0.0, 1.97)	0.2 (0.0, 0.6)
*J*_TA,STN_	1.7 (0.7, 2.6)	1.4 (0.5, 2.3)
*J*_D1,CTX_	7.8 (0.4, 13.4)	2.7 (0.0, 7.4)
*J*_D2,CTX_	2.6 (0.0, 10.6)	9.7 (7.3, 12.0)
*J*_FSI,CTX_	1.9 (0.0, 6.4)	8.9 (4.4, 13.2)
*J*_STN,CTX_	3.8 (1.5, 6.4)	0.83 (0.0, 2.6)

Our results also predict that cortical projections to FSIs are stronger in parkinsonian networks (physiological: *J*_FSI,CTX_ = 1.9, parkinsonian: *J*_FSI,CTX_ = 8.9). This prediction, however, is in contradiction with the studies like Wiltschko et al. (2010), Clarke and Adermark (2015) which show that dopamine agonists increase FSI activity due to presence of D1 like D5 receptors.

Experimental data on the effective strengths of projections emanating from and projecting to GPe-TA-TI has been largely missing, until recently. In one such study, Glajch et al. (2016) measured IPSCs in *ex vivo* slices from two types of GPe neurons, one expressing NPas1^+^ and other expressing PV^+^. Neurons expressing NPas1^+^ correspond to arkypallidal (GPe-TA) neurons and those expressing PV^+^ correspond to prototypical (GPe-TI) neurons, as also shown by Abdi et al. (2015). It turns out that several relationships between the effective strengths of GPe-TA-TI connections in physiological and parkinsonian conditions observed by Glajch et al. (2016) can also be found in our generated ensembles:

- The projections from GPe-TA to D2-MSNs are stronger than those to D1-MSNs. Moreover, both of these projections are stronger in in 6-OHDA conditions
  (physiological: *J*_D2,TA_ = −1.2, *J*_D1,TA_ = −0.83; parkinsonian: *J*_D2,TA_ = −1.4, *J*_D1,TA_ = −0.93).
- The projections from GPe-TA population to striatal MSNs (see above) are stronger than the corresponding projections from GPe-TI population; this is also consistent with the observations in Mallet et al. (2012) and Abdi et al. (2015).
  (physiological: *J*_D2,TI_ = −0.2, *J*_D1,TI_ = −0.3; parkinsonian: *J*_D2,TI_ = −0.6, *J*_D1,TI_ = −0.18).
- GPe-TI projections to D2-MSNs increased in parkinsonian conditions
  (physiological: *J*_D2,TI_ = −0.2; parkinsonian: *J*_D2,TI_ = −0.6)
- GPe-TI projects more strongly to FSIs than GPe-TA
  (physiological: *J*_FSI,TI_ = −0.82, *J*_FSI,TA_ = −0.23; parkinsonian: *J*_FSI,TI_ = −1.0, *J*_FSI,TA_ = −0.23)
- GPe-TI projects more strongly to STN than GPe-TA in naive mice; this is also consistent with the observations in Mallet et al. (2012) and Abdi et al. (2015) that GPe-TI mostly projects to downstream nuclei in basal ganglia.
  (physiological: *J*_STN,TI_ = −2.0, *J*_STN,TA_ = −0.75).

Only one prediction is in contradiction with Glajch et al. (2016), which predicts that GPe-TI projections to D1-MSNs remain unchanged when measured in naive and 6-OHDA lesioned mice, whereas our distributions show a slight decrease in GPe-TI projections to D1-MSNs in parkinsonian conditions (*J*_D1,TI_ = −0.18) as compared to physiological conditions (*J*_D1,TI_ = −0.3). Predictions of the relationships that go beyond current experimental observations are listed and discussed in Section 4.2. In total, the good fit of the generated structural relationships in our network ensembles to experimentally observed relationships from a variety of sources allow us to conclude that these structural relationships are crucial to evoke the different dynamics in the physiological and parkinsonian conditions.

### 3.3. Variability and correlations in the effective connectivities for physiological and parkinsonian networks

**Figure 4:**
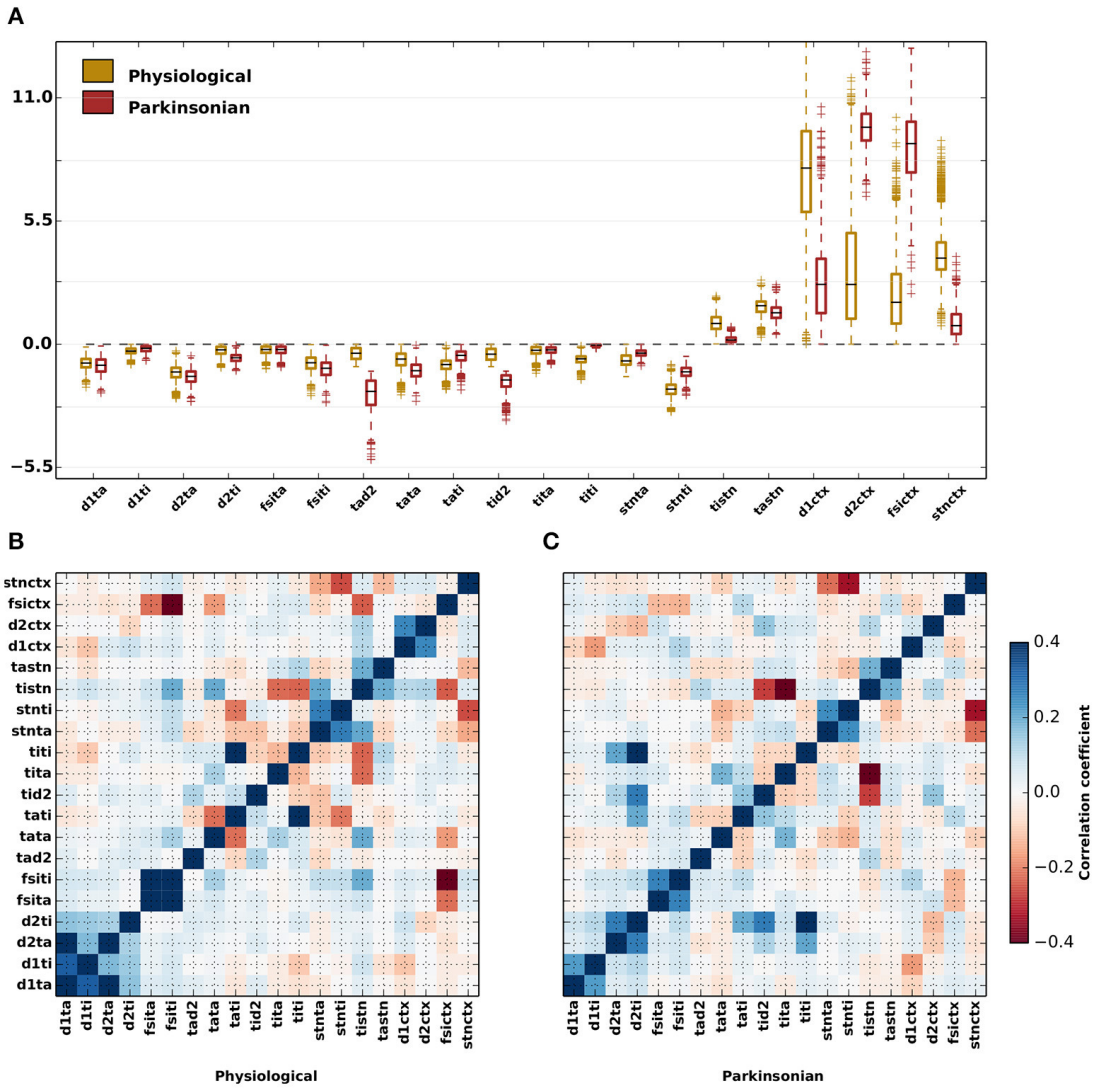
Variability of parameters for parkinsonian and physiological network configurations. **(A)** Distributions of values generated by the genetic algorithm for each of the free parameters displayed as box plots for physiological (yellow) and parkinsonian (red) networks. The black lines within the box plots represent the median values, whereas the box caps represent the 25% (Q1) and 75% (Q3) quartiles of the distributions. The whiskers represents the margin: (Q1–1.5×IQR, Q3+1.5×IQR), where IQR = Q1 − Q3. Outliers to the margins are represented by “+” markers. The values of the medians and quartiles are listed in Table 5. **(B)** Correlation coeffiecients between the free parameters for physiological networks **(C)** As in **(B)** but for parkinsonian networks.

The effective connection strengths generated by the genetic algorithm show a considerable variation across the ensemble of 1,214 physiological and 1,265 parkinsonian network configurations, respectively (Figure 4A and Table 5). Whereas some parameters have a narrow distribution (e.g., *J*_D1,TI_ for both categories, *J*_TI,TI_ and *J*_TI,STN_ for parkinsonian networks), others are broadly distributed (e.g., *J*_TA,D2_ for parkinsonian networks, *J*_D1,CTX_ and *J*_FSI,CTX_ for both categories). Moreover, pairs of effective connection strengths are typically correlated (Figures 4B,C). Both in physiological and parkinsonian networks, a number of effective-weight pairs are positively correlated (e.g., {*J*_D1,TA_, *J*_D1,TI_}, {*J*_FSI,TA_, *J*_FSI,TI_}, {*J*_TA,STN_, *J*_TI,STN_},{*J*_D1,CTX_, *J*_D2,CTX_}), others exhibit negative correlations (e.g., {*J*_TA,TI_, *J*_TA,TA_}, {*J*_TI,TA_, *J*_TI,TI_},{*J*_TI,TA_, *J*_TI,STN_}). For most connection pairs, the magnitude of correlations is slightly smaller in parkinsonian networks. Exceptions are, for example, strong positive correlations for {*J*_TI,TI_, *J*_D2,TI_} and {*J*_TI,TI_, *J*_D2,TA_} pairs in parkinsonian networks, which are not observed in the physiological case. Further, {*J*_TI,STN_, *J*_TI,D2_} and {*J*_TI,STN_, *J*_TI,TA_} pairs are more negatively correlated in parkinsonian networks as compared to the physiological case.

For some projections, the difference between the physiological and parkinsonian distributions is striking, for example the much reduced cortical projection strength to D1-MSNs *J*_D1,CTX_. The distributions of other connections, e.g., inhibition from GPe-TA to GPe-TI, *J*_TI,TA_, are more similar.

**Figure 5:**
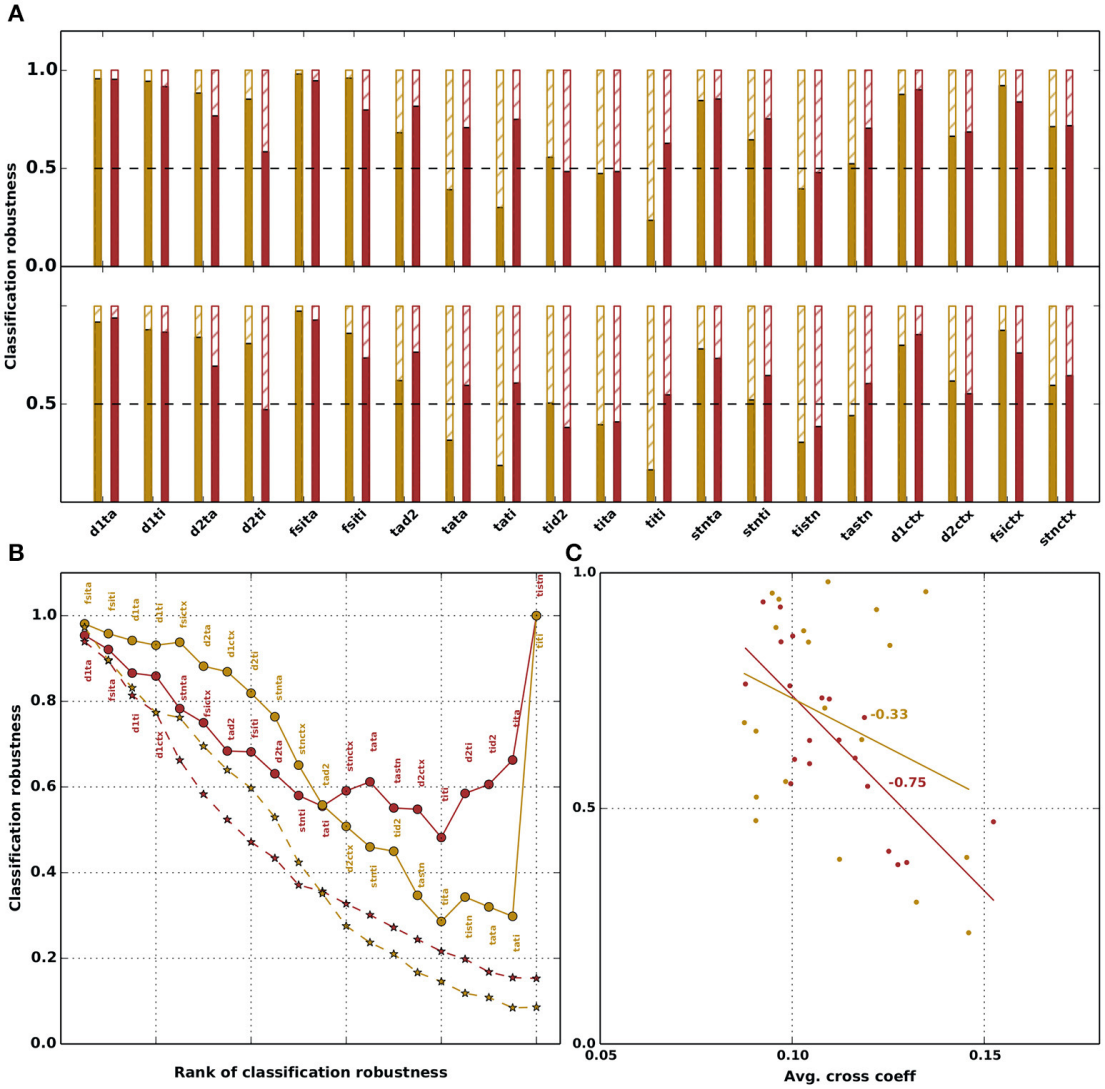
Robustness of network classification for physiological (yellow) and parkinsonian (red) networks with respect to manipulation of the parameters. **(A)** Classification robustness, i.e., fraction of networks that retain (solid bars) or lose (hatched bars) their respective classification under manipulation of each free parameter. Top row: parameter value is replaced by the mean of the corresponding distribution (*replace by mean*). Bottom row: parameter values are randomly shuffled within the ensembles of network configurations with the same classification (*shuffle*); classification robustness is averaged over ten shuffle realizations. **(B)** Classification robustness as a function of the number of parameters manipulated in a cumulative fashion. Circles: *replace by mean*. Stars: *shuffle*. Order of manipulations is given by decreasing order of classification robustness in (A).This order is fixed for the *replace by mean* manipulation (see annotations next to circles); for the *shuffle* manipulation (stars), the order depends on the realization of the random shuffle. Stars depict robustness averaged over ten shuffle realizations. **(C)** Classification robustness as a function of the mean correlation coefficient of each free parameter (from matrices shown in Figures 4B,C). A line fit shows a strong dependence with a *R*-value of −0.75 for parkinsonian networks and a weak dependence with *R*-value of −0.33 for physiological networks.

To evaluate the functional significance of the observed variability and covariability in the effective connectivity, we test whether the classification of networks into “physiological” and “parkinsonian” networks according to the criteria described in Table 4 is robust with respect to (i) replacing effective connection weights by the mean for that connection across the ensemble (Figure 5A, top row), or (ii) shuffling the weights across network configurations in the same ensemble (Figure 5A, bottom row). The classification robustness is defined as the fraction of networks that retain their original classification after a given manipulation. The two manipulations are described in further detail in Section 2.4.

The *replace by mean* manipulation tests the assumption that the distribution of a parameter is just fluctuations around a mean and is thus not important for the dynamics of the network. The *shuffle* manipulation allows us to investigate whether it is sufficient for the network dynamics to preserve the marginal weight distributions across network configurations. Both types of assumptions are very common in modeling studies integrating experimentally obtained parameters, and neglect correlations between the effective weights.

For the majority of connections, replacing the weight by the corresponding ensemble average preserves the original classification for more than 50% of both physiological and parkinsonian networks. For some cases the classication robustness is very high, for instance, replacing the weight *J*_D1,TA_ of the GPe-TA inhibition to D1-MSNs by the ensemble average preserves the classification of about 95% of networks. However, for a number of connections (e.g., *J*_TI,STN_), replacing the weight by the ensemble average causes a substantial fraction of networks (more than 50%) to no longer exhibit the dynamics described in Table 4 for their original classifications. Similar results are obtained by shuffling weights across the ensemble of network configurations (bottom panel in Figure 5A). Overall, the classification robustness is lower in this case.

The above analysis is based on replacing the weight of individual connections, leaving the other connections unaltered. It is not clear whether manipulations of one parameter affect the robustness with respect to other parameters. To address this, we repeat the analysis in a cumulative fashion, i.e., performing the manipulations for each parameter in turn without resetting the values changed in the previous manipulation. The parameters are manipulated in order of decreasing classification robustness in the individual case, as shown in Figure 5A. For the *replace by mean* manipulation, this order is fixed, and shown as annotations to the data points (Figure 5B, solid curves). The *shuffle* manipulation is carried out ten times to test the robustness of the results with respect to different random weight realizations. Potentially, each of these ten realizations has a distinct classification-robustness order. The dashed curves in Figure 5B depict averages across the ensemble of ten realizations.

With this ordering, a cumulative replacement of weights by their respective ensemble averages leads to a maximum reduction of classification robustness to about 0.3 for physiological networks and 0.5 for parkinsonian networks, although initially the physiological networks are more robust with respect to multiple manipulations. Counterintuitively, if all weights are replaced by their ensemble averages (rightmost circles in Figure 5B), the fraction of unchanged networks jumps back to 1. This suggests that the correlations observed between the parameters in Figures 4B,C are not artifactual, but are necessary for producing the dynamics of the two categories. By restricting some parameters to their means, but allowing others to maintain their distributions, some of the resulting network configurations fall outside of the volume in 20-dimensional parameter space that contains the original ensemble, and no longer exhibit the correct dynamics. As more parameters are restricted, the effect increases. However, once all parameters are set to the means of their respective distributions, the resulting network configuration is within the original volume, and thus retains the original classification. A similar effect can be seen with the *shuffle* manipulation, which transforms an n-dimensional ellipsoid of network configurations into an n-dimensional sphere, thus also causing many network configurations to be shifted to points outside the original volume. Here, the classification robustness decays monotonically.

To quantify the relationship between correlation and classification robustness, in Figure 5C we plot the fraction of networks that retained their classification for each parameter against the average cross correlation coefficient between that parameter and the others, calculated from the matrices shown in Figures 4B,C. A strong dependency for parkinsonian and a weak dependency for physiological networks is evident, with a line fit yielding an *R*-value of −0.75 for parkinsonian and −0.33 for physiological networks.

In total, these results demonstrate that there is a wide variability in the parameter configurations that pass the selection criteria and can reproduce the network activity shown in Figure 2. Our results show the dangers of restricting the parameter search to produce a single set of parameters, especially by taking the mean of distributions, unless this is carried out systematically for all distributions. Similarly, our results illustrate that the variability in parameter configurations cannot be boiled down to the marginal distributions of the individual parameters, due to presence of correlations between them.

### 3.4. Physiological and parkinsonian networks form distinct clusters in the dynamical feature space

In order to investigate the functional consequences of the variability of the generated solutions shown in Figures 4, 5, we evaluated the network configurations on the basis of their dynamical properties, identified from experimental observations on basal ganglia activity in physiological conditions and Parkinson's disease. It has been shown that insufficient GPi suppression is usually associated with stymied movement. For example, Boraud et al. (2000) showed that the ratio of inhibited-to-activated GPi neurons is significantly reduced during movement in a MPTP-treated monkey. In an another experiment, the delayed suppression of GPi neurons was shown to be correlated with slowing down of movement (Leblois et al., 2006). Akinesia, which is the loss or impairment of voluntary movements, is also associated with oscillations in the basal ganglia system. The akinetic symptoms of the Parkinson's patients have been shown to grow worse with an imposed low-frequency stimulation of 10–20 Hz in STN (Timmermann et al., 2004; Chen et al., 2007). A tACS (transcranial alternating stimulation) of 20 Hz in cortex leads to slowing of movements in healthy individuals (Pogosyan et al., 2009).

Another parkinsonian symptom associated with the oscillation frequency of basal ganglia, specifically the GPe, STN, GPi and thalamus, is tremor. Electrophysiological studies have found single units oscillating at tremor frequencies in STN and pallidum (Raz et al., 2000; Levy et al., 2002) as well as oscillations of large populations in STN oscillating at 8 − 20Hz in tremor-dominant Parkinson's patients (Moran et al., 2008). These dynamical features also reflect the effect of striatal bias downstream in the basal ganglia. A lack of striatal bias toward “Go” can contribute to insufficient suppression of GPi rates. Moreover, we previously showed in a numerical study that an excess of striatal bias toward “No-Go” can initiate oscillations in the GPe-STN circuit (Kumar et al., 2011).

We therefore evaluate the networks' responses to a transient square pulse with respect to two dynamical features, namely the effective suppression of GPi rates to a transient cortical activity (GS) and the susceptibility of basal ganglia circuit to oscillations (SO). It should be noted that this input was not used for parameter fitting in genetic algorithms to distinguish between parkinsonian and physiological networks; the response to this input is an emergent feature of the effective connectivities generated to fulfill the parkinsonian or physiological criteria given in Table 4. The GPi suppression (GS) is calculated as the normalized difference of GPi rates in presence and absence of the cortical activity, such that a value of one for GS represents efficient suppression of GPi and zero or negative values indicate ineffective suppression of GPi rates. The susceptibility to oscillations (SO) is calculated from the spectral entropy (SE). A white noise signal, for example, will show a power spectrum with no clear peak and hence high entropy, whereas for an oscillatory signal, the power will be concentrated in a certain frequency band, yielding a lower entropy. We define the susceptibility to oscillations as (1 − SE), such that values toward zero indicate low SO (high SE) and values toward one indicate high SO (low SE). A full definition of both measures and the details of the transient cortical input used as stimulation are given in Section 2.3.

**Figure 6:**
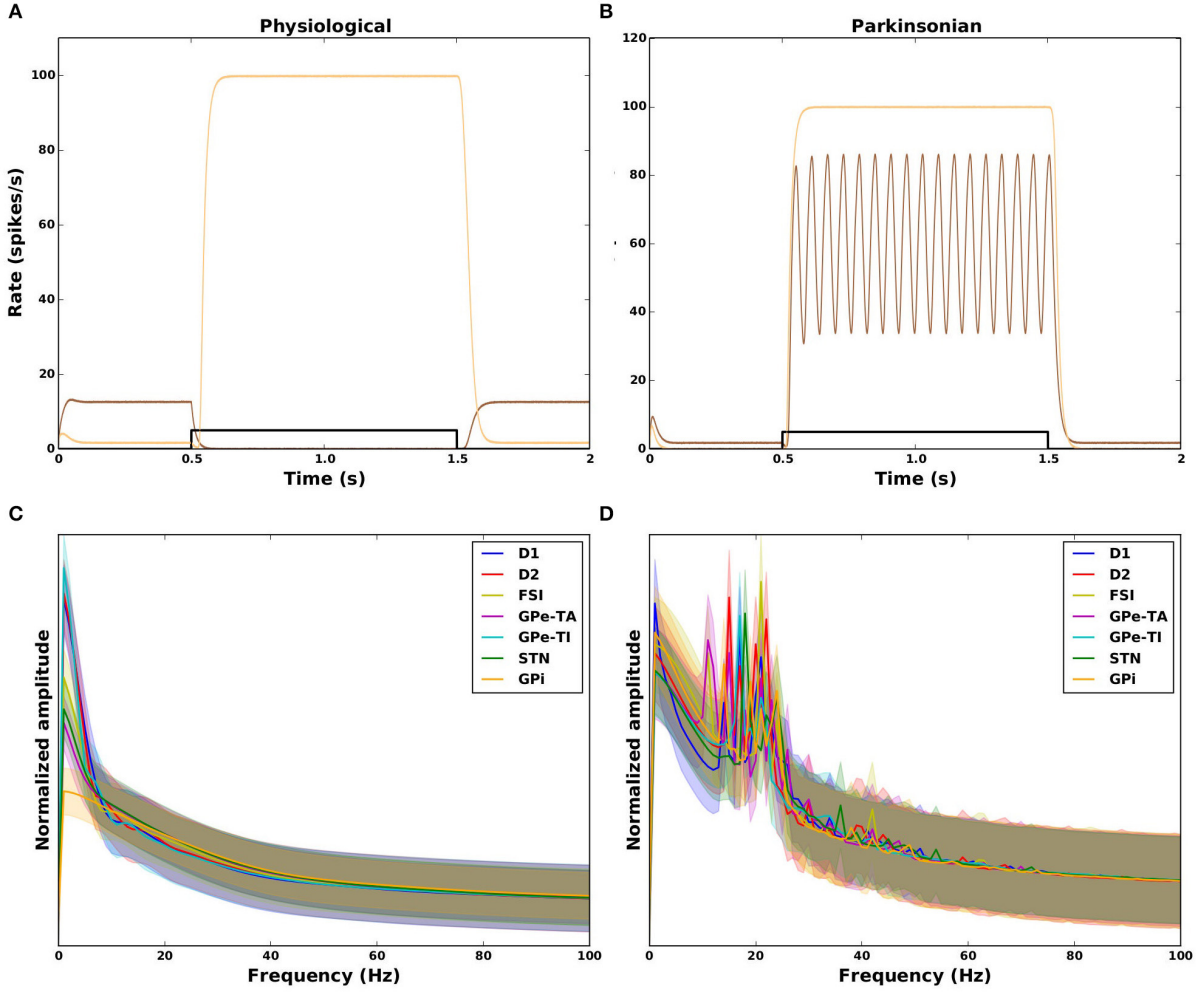
Dynamical features of physiological (left column) and parkinsonian networks (right column). Top row: response of the GPi to a 1s square pulse from the cortex (black). **(A)** Two example physiological networks (brown: GS = 1.0, SO = 0.03, orange: GS = −0.93, SO = 0.09). **(B)** Two example parkinsonian networks (brown: GS = −0.93, SO = 0.93, orange: GS = −0.9, SO = 0.03). **(C, D)** Amplitude spectrum (normalized by area) of activity in response to the transient square pulse for all nuclei in the model. Thick curves: mean across network configurations. Shaded areas: mean±standard deviation across network configurations. Frequency resolution: 1 Hz.

An illustration of the relationship of these measures to the activity evoked in the GPi by a transient square pulse is given for some example networks in Figures 6A,B. The physiological networks (Figure 6A) show no oscillatory activity (SO = 0.03 and SO = 0.09) with either effective (brown; GS = 1.0) or ineffective suppression of GPi activity (orange; GS = −0.93). The two examples of parkinsonian networks in Figure 6B both show an increase in the GPi activity (hence ineffective GPi suppression) during the cortical transient input (shown in black), the corresponding values for GS are therefore negative (−0.9 and −0.93). One example shows strong oscillatory activity (brown; SO = 0.93) whereas the other shows no oscillations (orange; SO = 0.03). The absence and presence of oscillatory activity in physiological and parkinsonian networks, respectively, can also be seen in the amplitude spectrum plotted in Figures 6C,D. The mean normalized spectrum (thick curves) for parkinsonian networks (Figure 6D) shows a clear peak in the beta range (15–25 Hz) for all nuclei, whereas the mean spectrum for physiological networks shows no such peak (Figure 6C).

**Figure 7:**
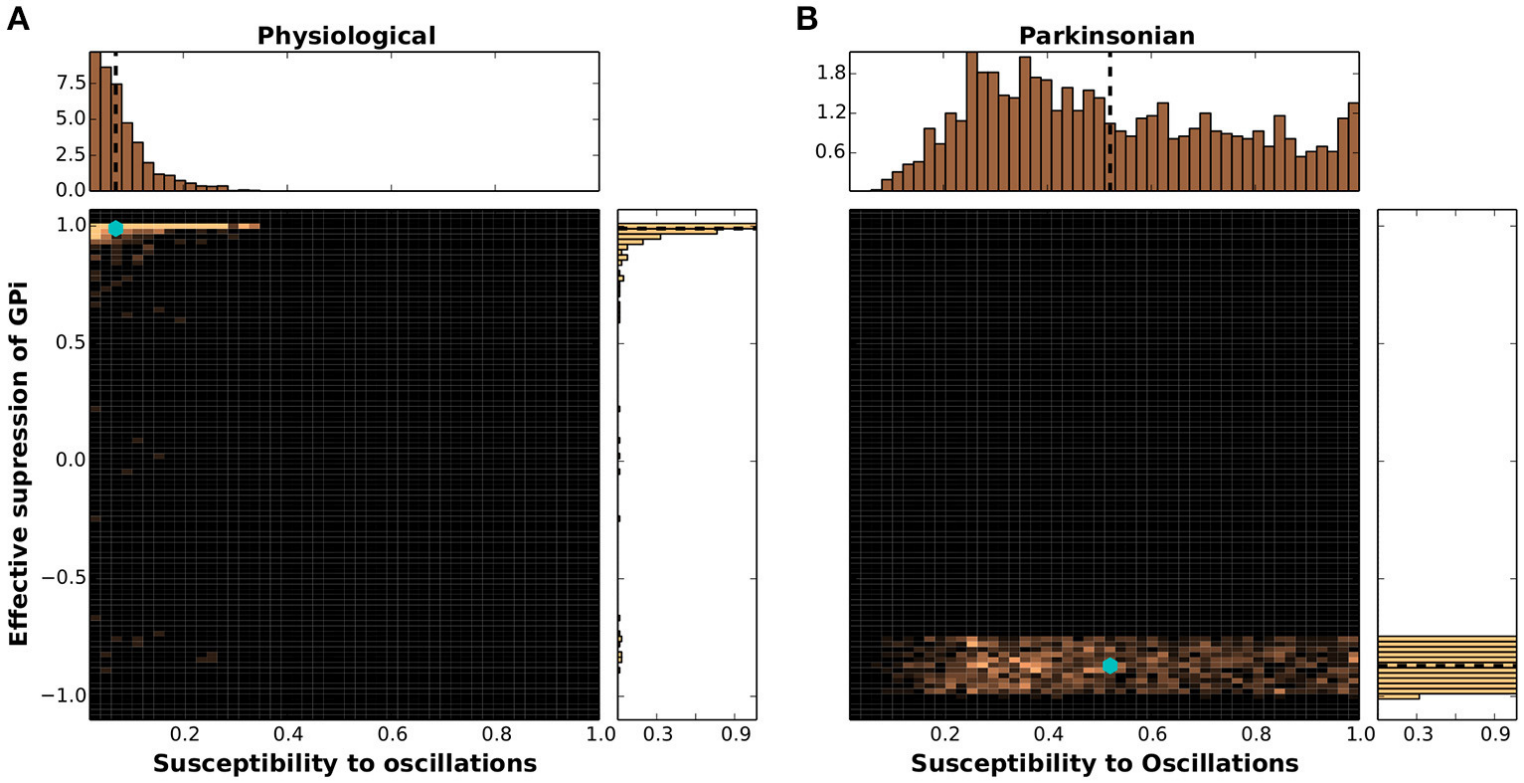
Clustering of networks in dynamical feature space. **(A)** 2D histogram of physiological networks projected into the feature space spanned by susceptibility to oscillations (SO) and GPi suppression (GS), mean indicated by a cyan marker. Margins give the corresponding 1D histograms, with means indicated by black dashed lines. **(B)** As in **(A)** but for parkinsonian networks.

The results of projecting the network configurations classified as physiological and parkinsonian onto the dynamical feature space defined by SO and GS is given as 2D histograms in Figure 7. The physiological networks displayed in Figure 7A have a mean at SO = 0.06, GS = 0.97 (indicated by a cyan marker), which indicates low susceptibility to oscillations and sufficient suppression of GPi activity. The distribution of SO values is well fit by a gamma distribution with shape parameter *k* = 1.48, scale parameter θ = 0.045 and a mean of 0.06. The distribution of GS values is well fit by a power law, with parameter α = 45 with a mean of 0.97. The parkinsonian networks (Figure 7B) show a mean at SO = 0.53, GS = −0.86, indicating a much higher susceptibility to oscillations and insufficient suppression of GPi activity. Unlike the physiological networks, in this case a closer look at the 1D histograms reveals distributions of values that are well fit by a triangular distribution for SO with lower limit *a* = 0.07, upper limit *b* = 1.07 and mode *c* = 0.3, and a uniform distribution for GS between −1.0 and −0.75 but also exhibiting a small scattering of outliers reaching up to much higher values.

These results demonstrate clearly that although the physiological and parkinsonian networks generated by the genetic algorithm exhibit large variability in the 20-dimensional structural space, they cluster together in the 2-dimensional dynamical feature space and exhibit an unambiguous separation between networks classified as physiological and those classified as parkinsonian. Most of the parkinsonian networks show low GS and high SO (Figure 7B, bottom), whereas most of the physiological networks show high GS and low SO (Figure 7A, top left).

However, there is some overlap between the physiological and parkinsonian networks (e.g., bottom left corner, SO ≈ 0.1 and GS ≈ −0.9). This could indicate that these two dynamical features are not enough to disambiguate these parkinsonian and physiological networks. However, it may also turn out that some networks are barely distinguishable even when projected onto a space of many such functional features. Such networks might be particularly useful for giving insight into the transition from physiological state to parkinsonian and vice versa.

It should also be noted, that although the homologies were constrained based on the characteristics of GPe(TA/TI)-STN subnetwork, they show expected dynamical features that depend on the activity of the entire BG network. That is, 70% of parkinsonian networks show a GS < −0.5 and a SO > 0.35, whereas 95% of physiological networks show GS > 0.8 and SO < 0.2. This indicates that the network characteristics of the BG subcircuit (GPe-STN) such as firing rate and phase relationships as shown in Mallet et al. (2008) and Abdi et al. (2015) are predictive of the network state of the entire BG in GS-SO space.

## 4. Discussion

In this study, we used a genetic algorithm to perform a search over 20 free effective connectivity parameters, generating thousands of basal ganglia network configurations that fulfill the firing rate and phase relationships reported by Mallet et al. (2008) and Abdi et al. (2015) for physiological and parkinsonian networks. Although relationships between effective connectivity strengths were not included in our classification criteria, we observe a very good fit of many of the structural relationships exhibited by our generated ensembles of networks to experimental data. We observed that both the breadth of the distribution of the individual parameters and the degree of overlap in the distributions between physiological and parkinsonian distributions were subject to substantial variation.

To determine whether the variance for individual parameters was simply a side effect of our method of generation, and could therefore be reduced, we replaced the distributions by their means and checked what proportion of the networks retained their physiological or parkinsonian classification (i.e., according to the criteria listed in Table 4). For most connections, replacing the weight by the corresponding ensemble average preserves the original classification for at least 50% of both physiological and parkinsonian networks. A similar effect was observed when parameter values were shuffled between members of the same ensemble.

When the replace-by-mean manipulation was performed cumulatively, the classification robustness fell until it saturated at about 0.3 for physiological networks and 0.5 for parkinsonian networks. However, when all parameters were replaced by their means, the remaining networks retained the correct classification. Cumulative application of the shuffle manipulation resulted in a monotonic decrease in robustness. We interpret these results as indicating that the ensembles generated by the genetic algorithm give a good approximation of the 20-dimensional volume in the parameter space that produces the correct dynamics for the physiological and parkinsonian conditions. Parameter manipulation that take a network configuration out of this space tend to result in the network no longer exhibiting the corresponding dynamics. Further, these results demonstrate that the correlations between the parameters are necessary for producing the dynamics associated with the two categories. Beyond the current study, this suggests that modelers should be cautious in taking an approach in which some parameters are allowed to be heterogeneous whereas others are restricted to mean values; networks constructed in this fashion may be less representative of the target system than a more reduced, simpler model where all parameters are fixed to mean values.

We projected the 20-dimensional space spanned by connection weights to a 2-dimensional space of dynamical features defined by the response of a network to a transient cortical pulse, namely: (1) its capacity to suppress GPi activity (GS), and (2) its susceptibility to oscillations (SO). Our results show that despite the high variability and overlap in the 20-dimensional structural space, solutions form distinct physiological and parkinsonian clusters in the dynamical space. Most of the networks classified as parkinsonian show ineffective suppression of GPi activity (low GS) and high susceptibility to oscillations (high SO). Conversely, the majority of physiological networks show effective suppression of GPi activity (high GS) and low susceptibility to oscillations (low SO). This suggests that the characteristics of the BG subcircuit (GPe-STN) as shown in Mallet et al. (2008) and Abdi et al. (2015) are predictive of the network state of the entire BG and can serve as viable biomarkers of the network state.

It should be emphasized that the response of the network to the transient input used to measure GS and SO was not used in the parameter search, or to classify the networks as physiological or parkinsonian. The emerging dynamical features (GS and SO) are not a trivial consequence of the constraints in Table 4. For example, due to the complex recurrent interplay between all nuclei, the absence of cortical modulation of GPe in physiological conditions [constraints (7) and (9)] does not directly imply a suppression of the GPi response. Similarly, due to the constraints (6)–(10) in Table 4, one may expect an increased susceptibility of the network to SWA oscillations at 2 Hz, but not in the beta range at 20 Hz (cf. peaks in spectra in Figure 6D).

In the rest of this sections we briefly delineate the limitations arising from our approach on interpreting our results, before discussing novel predictions of our study and the implications of the existence of the homologous networks generated by our parameter search.

### 4.1. Limitations

The model used here is a firing rate model, which is a highly simplified formulation that captures solely the evolution of the mean activity of a population. This kind of model inherently limits the insights that can be gained about the dynamics of the system, for example it can give no information about the structure of individual spike trains or higher order correlations within a population. However, it has the advantage of being computationally less expensive and with a restricted parameter space with respect to higher resolution simulation methods (e.g., spiking neuronal networks). It should be noted, that an another modeling work has described a spiking neural network model of the basal ganglia by including the GPe-TA/TI subpopulations (Lindahl and Hellgren Kotaleski, 2016). Although, the model description of the aforementioned work is more detailed (spiking neural networks) as compared to mean field models in our case, the former constrains the system to exactly one solution of effective weights. It would be interesting to check if these effective weights falls under the solution space spanned by the homologies in this work.

In this study we considered those parameters, for which experimental data sets exist, to be fixed. However, in Section 3.3 we discovered that replacing the generated values for the free parameters with the mean of the corresponding distribution does not necessarily preserve the dynamical properties of the networks, at least in part due to correlations between the free parameters. This argument could be equally well applied to the “fixed” parameters. A better approach might be to consider the fixed parameters as free, albeit within the biological range suggested by the experimental data. We plan to incorporate this extension in the future work. This extension will enables us to address changes in effective connectivities in the fixed parameters such as increase in the inhibition from FSI to D2 (Gittis et al., 2011; Corbit et al., 2016) and decrease in striatal lateral inhibition during parkinsonian conditions (Taverna et al., 2008) which are expected to be emergent trends in the weight distributions or can be used as additional *post-hoc* constraints to reduce the number of homologies.

Ideally one would choose the parameters of the activation function according to F-I curves obtained in *in-vitro* experiments. However, the activation function *S*(·) used in the model corresponds to the stationary firing rate response of an entire nucleus, rather than a single neuron. One could consider the parameters of the activation function as free parameters which are adjusted during the optimization process together with the synaptic weights. This would lead to a scenario where not only the synaptic weights but also the activation function parameters are widely distributed across network configurations. Future work can determine whether the additional insight gained by such an approach is sufficient to justify the increased computational complexity.

The cortical input in this model is considered as a feedforward input; the feedback connections from basal ganglia to cortex such as the pallidocortical (Chen et al., 2015) and thalamocortical projections are not included. Hence, the model cannot make predictions with respect to experimental observations such as pharmacological blocking of FSIs cause large negative suppressions of cortical LFPs which are time locked to fast involuntary movements (Klaus and Plenz, 2016). Only excitatory corticostriatal projections are considered, whereas recent data shows that somatostatin interneurons in the cortex also affect the striatal MSNs (Rock et al., 2016). The framework is sufficiently flexible to allow the inclusion of other projections and nuclei, however this would inevitably lead to an increase in number of free parameters.

The oscillations measured in our model in response to a square pulse of cortical input arise due to the change in the effective connectivities between the nuclei. However, oscillations might arise due to other causes, e.g., they may be purely delay driven. Since this model does not consider any delays, the results cannot provide any insight on the role of delays on the oscillations. Secondly, it has also been shown that the beta oscillations arise in striatum by increasing the activity of the striatal cholinergic interneurons pharmacologically (McCarthy et al., 2011). This model does not account for striatal cholinergic interneurons and make an alternative suggestion for the origin of oscillations in the absence of striatal cholinergic interneurons.

### 4.2. Predictions

In this study we collated experimental data on the strengths of connections within the basal ganglia to determine the fixed parameters of the model (see Section 2.1.1), and showed in Section 3.2 that the relationships between the distributions generated for the free effective connectivity parameters are a good match to experimental observations. On the basis of the connectivity strengths generated by our parameter search (see Table 5), we can also predict structural relationships for physiological and parkinsonian networks which are yet to be verified by experimental results:

- Striatopallidal projections are stronger in parkinsonian networks; this is consistent with the hypothesis that the indirect pathway becomes stronger in parkinsonian networks. Parkinsonian networks in our results also show a higher susceptibility to oscillations. These oscillations could initiate in the GPe-STN circuit and could be initiated by a strong indirect pathway, (i.e., a strong inhibition from striatum to GPe), as demonstrated in our recent numerical study (Kumar et al., 2011).
  (physiological: *J*_TA,D2_ = −0.4, *J*_TI,D2_ = −0.45; parkinsonian: *J*_TA,D2_ = −2.1, *J*_TI,D2_ = −1.6).
- Intrapallidal projections for GPe-TI are weaker in parkinsonian networks, but those for GPe-TA are stronger.
  (physiological: *J*_TI,TI_ = −0.64, *J*_TA,TA_ = −0.6; parkinsonian: *J*_TI,TI_ = −0.03, *J*_TA,TA_ = −1.2).
- GPe-TI-STN coupling is weaker in parkinsonian networks.
  (physiological: *J*_STN,TI_ = −2.0, *J*_TI,STN_ = 0.92; parkinsonian: *J*_STN,TI_ = −1.2, *J*_TI,STN_ = 0.2).

It is also noteworthy that these predictions are not a result of one model specification, but around thousand models that meet the firing rate and phase relationships to be considered physiological or parkinsonian. Therefore, firstly, we can state these predictions more confidently than if they had emerged from a fitting to a unique model specification, and secondly, as no limits on the structural relationships were placed on the parameter search, it seems reasonable to conclude that these emergent differences in these relationships for the physiological and parkinsonian ensemble are relevant for generating the differential dynamics of the two conditions.

We also checked these predictions under three different conditions: (a) increasing the inhibition from D2 to FSI (*J*_D2,FSI_) for parkinsonian networks; (b) keeping the projections from GPe-TA to STN (*J*_STN,TA_) fixed and set to zero; (c) including the projections from D1-MSNs to GPe (*J*_TA,D1_, *J*_TI,D1_) as free parameters. The parameters distributions for first two conditions are largely similar to the original distributions with no or few differences as described below.

- Experimental data shows that in parkinsonian networks, the FSI increase their connection to D2-MSNs (Gittis et al., 2011; Corbit et al., 2016). We checked this condition by increasing the fixed parameter *J*_D2,FSI_ given in Table 2 by a factor of 2.5 to −0.75, leaving *J*_D1,FSI_ fixed at its original value of −0.65. The resulting weight distributions (Supplementary Figure 3A) are qualitatively unchanged from those shown in Figure 4A, however some subtle alterations to the correlations between parameters can be observed (compare Figures 4B,C and Supplementary Figures 3B,C).
- For ensembles generated with *J*_STN,TA_ set to zero (Supplementary Figure 4), the distributions for most parameters are qualitatively similar to the original distributions (Figure 4A), except for D2 projections to GPe-TA (*J*_TA,D2_) and cortical projections to FSIs (*J*_FSI,CTX_). The former is weaker than D2 projections to GPe-TI(*J*_TI,D2_) in both physiological and parkinsonian networks, in contrast to the results shown in Figure 4, where they are approximately equal. Moreover, the projection *J*_FSI,CTX_ becomes weaker in parkinsonian than in physiological networks in contrast to the original ensembles where this finding is reversed.
- We also checked the predictions under the condition that projections from D1-MSNs to GPe (*J*_TA,D1_, *J*_TI,D1_) were also included as free parameters for the genetic algorithms. This inclusion showed significant qualitative changes in the parameter distributions (Supplementary Figure 5). Although some of the predictions are still consistent with the original distributions (Figure 4A), such as decrease in intrapallidal projections for GPe-TI (*J*_TI,TI_) in parkinsonian conditions, some predictions are at odds with the original model findings. For example, *J*_D2,CTX_ in physiological networks is approximately the same as *J*_D1,CTX_, whereas in the original distributions it is weaker.

In this work, we demonstrated that a particular limited set of features (Table 4) from the GPe-STN sub-circuit can predict dynamical characteristics (GS and SO) relevant for the behavior of the entire BG network under physiological and parkinsonian conditions. It remains to be investigated whether other feature sets have a similar predictive power and what the minimal set of predictive features is.

To test whether the mapping from the feature set in Table 4 to the dynamical characteristics (GS and SO) is one to one (injective), we performed a new set of numerical experiments where networks were constrained by the dynamical features GS and SO rather than by the original firing rates and phase relationships. We then checked whether the firing rate and phase relationships listed in Table 4 emerged as a result. Here, a transient square pulse was used as an input signal in the genetic algorithm. All the networks that showed GS ≤ −0.7 and SO ≥ 0.45 were classified as parkinsonian networks whereas the ones that showed GS ≥ 0.85 and SO ≤ 0.2 were classified as physiological networks. Only a fraction of the resulting networks are consistent with the criteria in Table 4 (Supplementary Figure 7). Hence, we can infer the dynamical feature GS and SO from the firing rates and phase relationships of GPe-TA-TI-STN subnetwork, but not vice versa. For the networks that fulfill the criteria in Table 4, the marginal weight distributions are very similar to the ones shown in Figure 4 (Supplementary Figure 6).

### 4.3. Homologous networks

Our parameter search led to over a thousand valid solutions (in 20 dimensional space) for both the physiological and the parkinsonian classification criteria. Although this could simply be a result of lack of constraints on the model, this could also indicate the presence of homologous networks in basal ganglia, in the sense that quite different parameter combinations can give rise to essentially the same dynamics. Homology has not yet been shown to occur in basal ganglia although other neuronal networks have shown occurrences of this phenomenon. However, it seems rather likely due to the complexity of the connectivity (Prinz et al., 2004).

What does the presence of homologous networks in our results imply? One possibility is that it is simply a consequence of the fact that we are mapping a high-dimensional parameter space (here network connections) onto a low dimensional space of network activity features (firing rates and phase relationships). Another possibility is that some parameters have only a small effect on the network activity features that we are interested in studying and can therefore be defined in a “sloppy” manner, i.e., with values are distributed over a large area in the parameter space (Gutenkunst et al., 2007). Our results show that indeed some parameters can be replaced by the mean or shuffled, whilst retaining the original classification for more than 95% of the ensemble. However, other parameters are much more sensitive to such manipulations. Thus, the existence of homologous networks could imply that that rather than absolute values of the parameters, it is their relative values that determine the dynamical state.

This interpretation is supported by the presence of correlations between the generated connectivity values, and the greater sensitivity of the dynamical system to those parameters that exhibit stronger cross-correlations (Figures 4B,C, 5C). The presence of correlations between the free parameters is also suggested as a possible reason for the presence of multiple solutions by Achard and De Schutter (2006). We propose that the approach of generating ensembles of homologous networks and analyzing them in terms of dynamical features is advantageous, as it gives us a framework for investigating complex interacting biological systems such as the basal ganglia which exhibit variability in structural parameters either due to nature (genetic tendencies) or nurture (plasticity in response to environmental factors).

In particular, it will enable us to examine whether the diversity shown by Parkinson's disease as a pathology is rooted in structural variability, to characterize the dynamic and structural properties of the transitions between different dynamical regimes, and uncover possible compensatory mechanisms. For example, we note that whereas the vast majority of physiological network configurations are located in a cluster characterized by effective suppression of GPi activity (high GS) and low susceptibility to oscillations (low SO), and parkinsonian networks typically display low GS and high SO, some network configurations do not fall into these clusters. Specifically, some networks classified as physiological with respect to their rates and phase relationships exhibit low GS, and some parkinsonian networks have low SO. These networks may simply be outliers, but they could also indicate the existence of transitional states between physiological and parkinsonian conditions.

Apart from the two suggested dynamical features (GS and SO), many others could be used to decompose a parkinsonian or physiological dynamical regime into more easily understandable dynamical features. Recent work by Escande et al. (2016) shows that the activity of D1-MSNs in response to optogenetic stimulation in cortex showed a gradient decrease from sham to partially lesioned 6-OHDA (and asymptomatic animals) to heavily lesioned (symptomatic animals). Hence, the gain of D1-MSNs could be yet another dynamical feature that could used for further functional classification.

On the basis of our results, we conclude that it may ultimately be more fruitful to study Parkinson's disease in terms of its dynamical features rather than its structural changes. Not only are dynamical features relatively low-dimensional, they also provide symptomatic targets for therapy (e.g., reduce susceptibility to oscillations) without presupposing specific underlying structures which may hold only in the mean, and not be applicable to a given patient.

## Author contributions

Conceived and designed the experiments: JB, TT, AK, AM. Performed the experiments: JB. Contributed reagents/materials/analysis tools: AK, TT, JH, AM. Contributed to the writing of the manuscript: JB, TT, AK, JH, AM.

### Conflict of interest statement

The authors declare that the research was conducted in the absence of any commercial or financial relationships that could be construed as a potential conflict of interest.

## Supplementary material

Supplementary Figure 1 Physiological and Parkinsonian weight distributions for three different random seeds.

Supplementary Figure 2 Firing rate distributions of physiological and parkinsonian homologies during cortical SWA and Beta input conditions. The histograms for each nuclei was calculated by pooling the activity (shown in Figure 3) across all parkinsonian and physiological networks.

Supplementary Figure 3 Parameters distributions when *J*_D2,FSI_ was increased by a factor 2.5 for parkinsonian networks. **(A–C)** same as Figures 4A–C.

Supplementary Figure 4 Parameters distributions when *J*_STN,TA_ was fixed to zero. **(A–C)** same as Figures 4A–C.

Supplementary Figure 5 Parameter distributions when connections from D1 to GPe-TA/TI are included as free parameters in the genetic algorithm. **(A–C)** same as Figures 4A–C.

Supplementary Figure 6 Parameter distributions when GPi suppression (GS) and Susceptibility to oscillations (SO) are used as constraints. **(A–C)** same as Figures 4A–C.

Supplementary Figure 7 Firing rates and phase relationships for valid and invalid networks when constrained using GS and SO. Gray lines: invalid networks. Cyan lines: Valid networks for GPe-TA. Green lines: Valid networks for GPe-TI. Compare with Figure 2.
