# Supplementary figures and images for: Homologous Basal Ganglia Network Models in Physiological and Parkinsonian Conditions
Source: Front Comput Neurosci. 2017 Aug 22;11:79. doi: 10.3389/fncom.2017.00079 (PMC5572265; doi:10.3389/fncom.2017.00079)

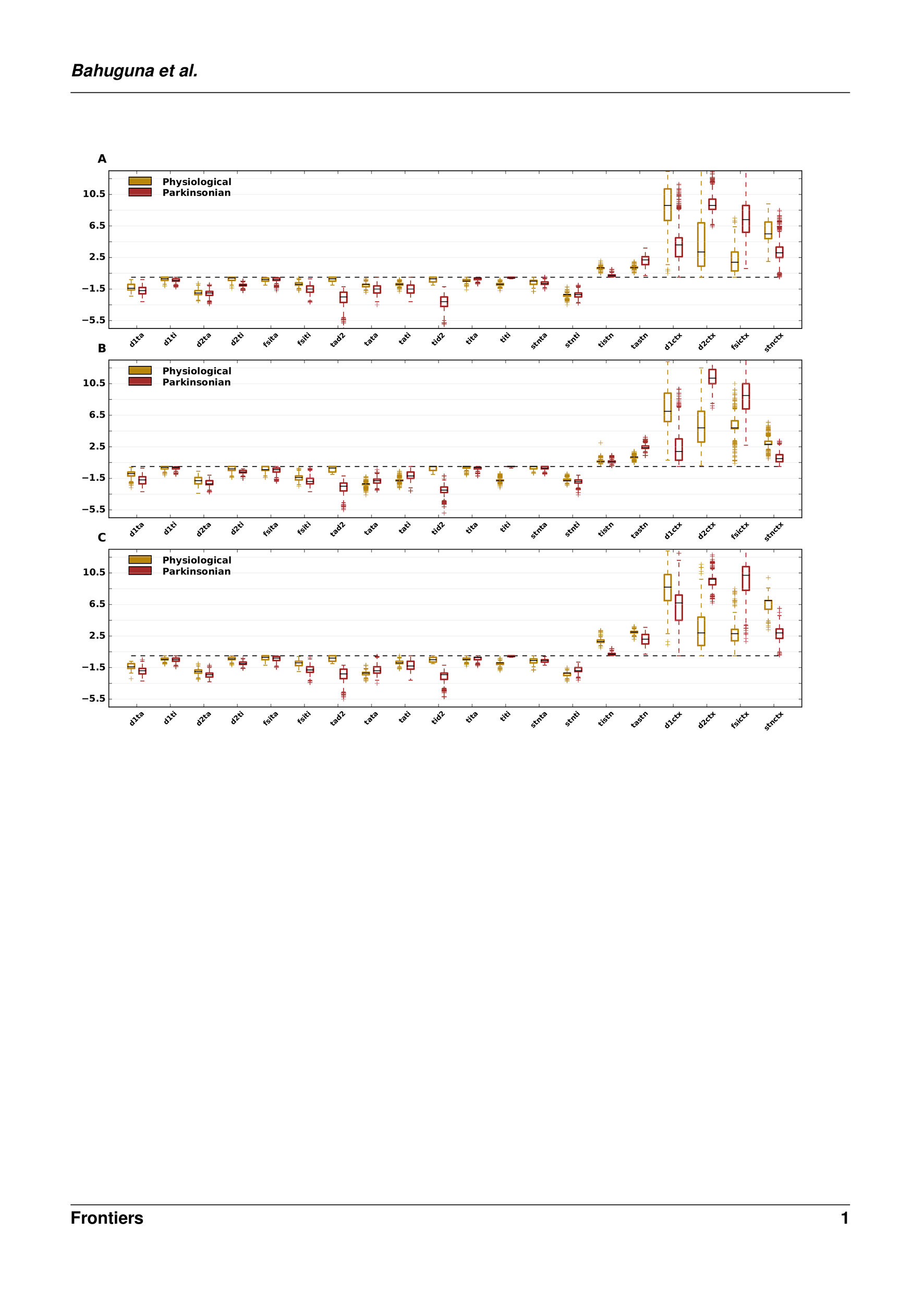

Supplement: Supplementary Figure 1 — Physiological and Parkinsonian weight distributions for three different random seeds. [file Image1.TIFF]

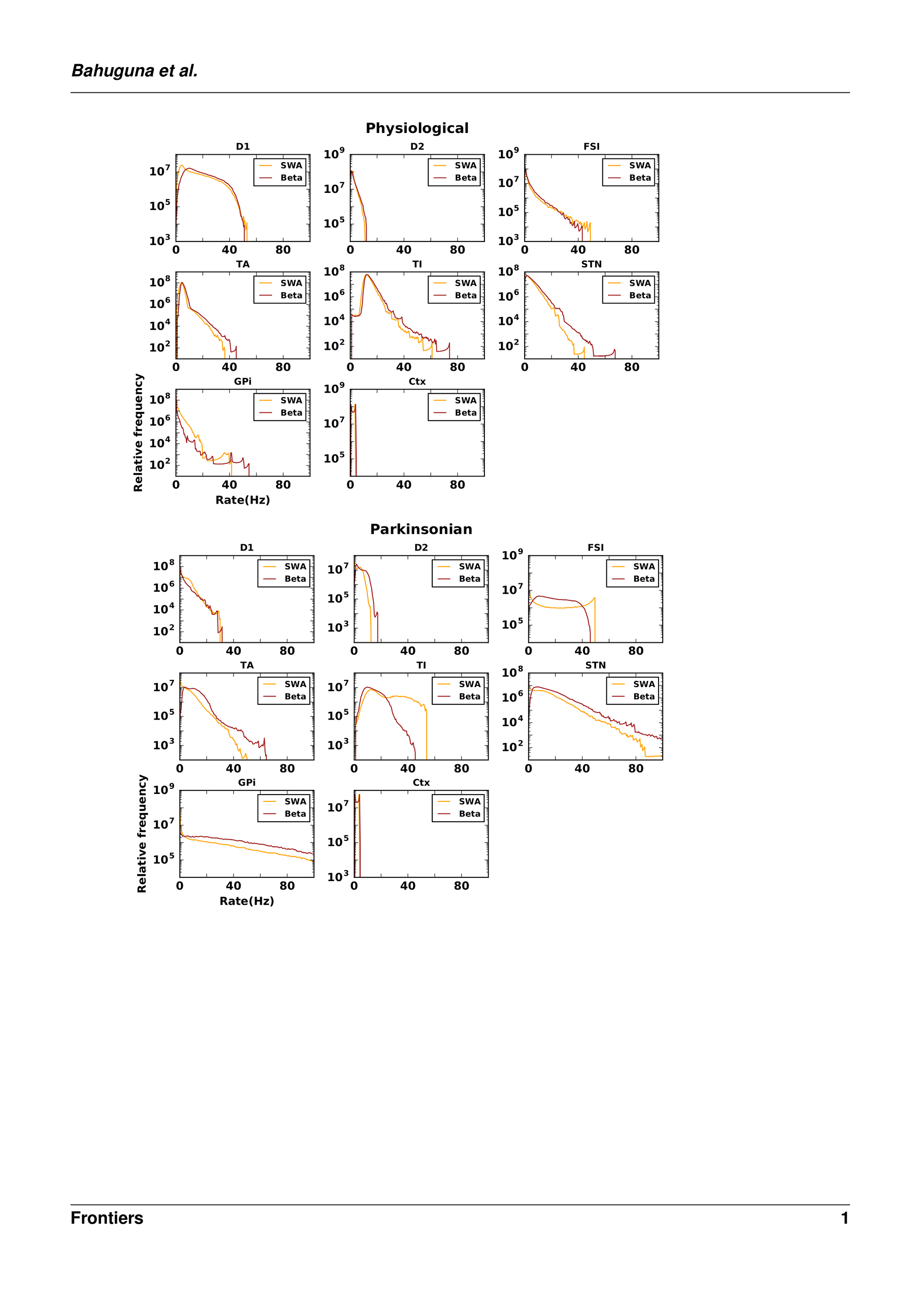

Supplement: Supplementary Figure 2 — Firing rate distributions of physiological and parkinsonian homologies during cortical SWA and Beta input conditions. The histograms for each nuclei was calculated by pooling the activity (shown in Figure 3) across all parkinsonian and physiological networks. [file Image2.TIFF]

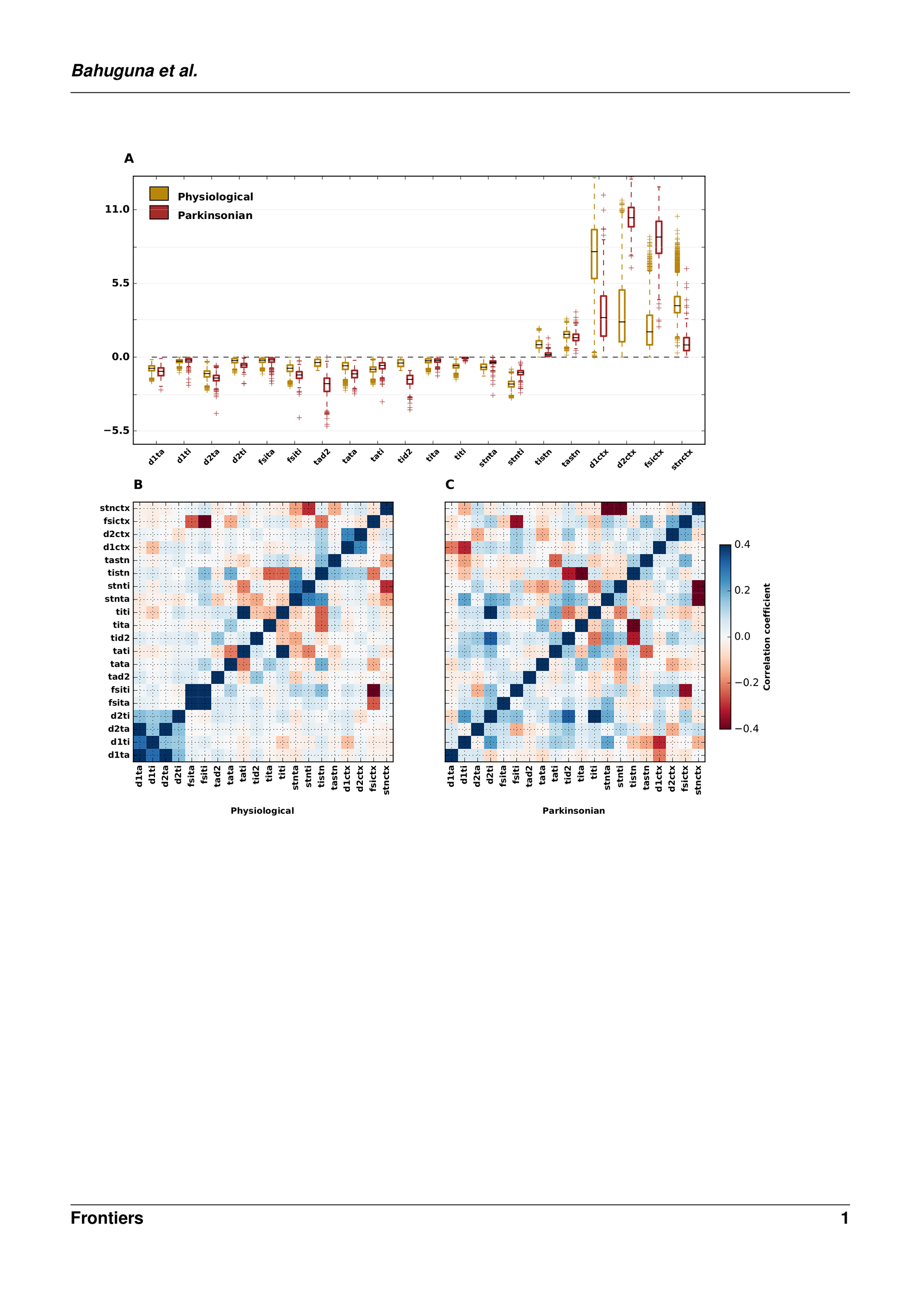

Supplement: Supplementary Figure 3 — Parameters distributions when JD2,FSI was increased by a factor 2.5 for parkinsonian networks. (A–C) same as Figures 4A–C. [file Image3.TIFF]

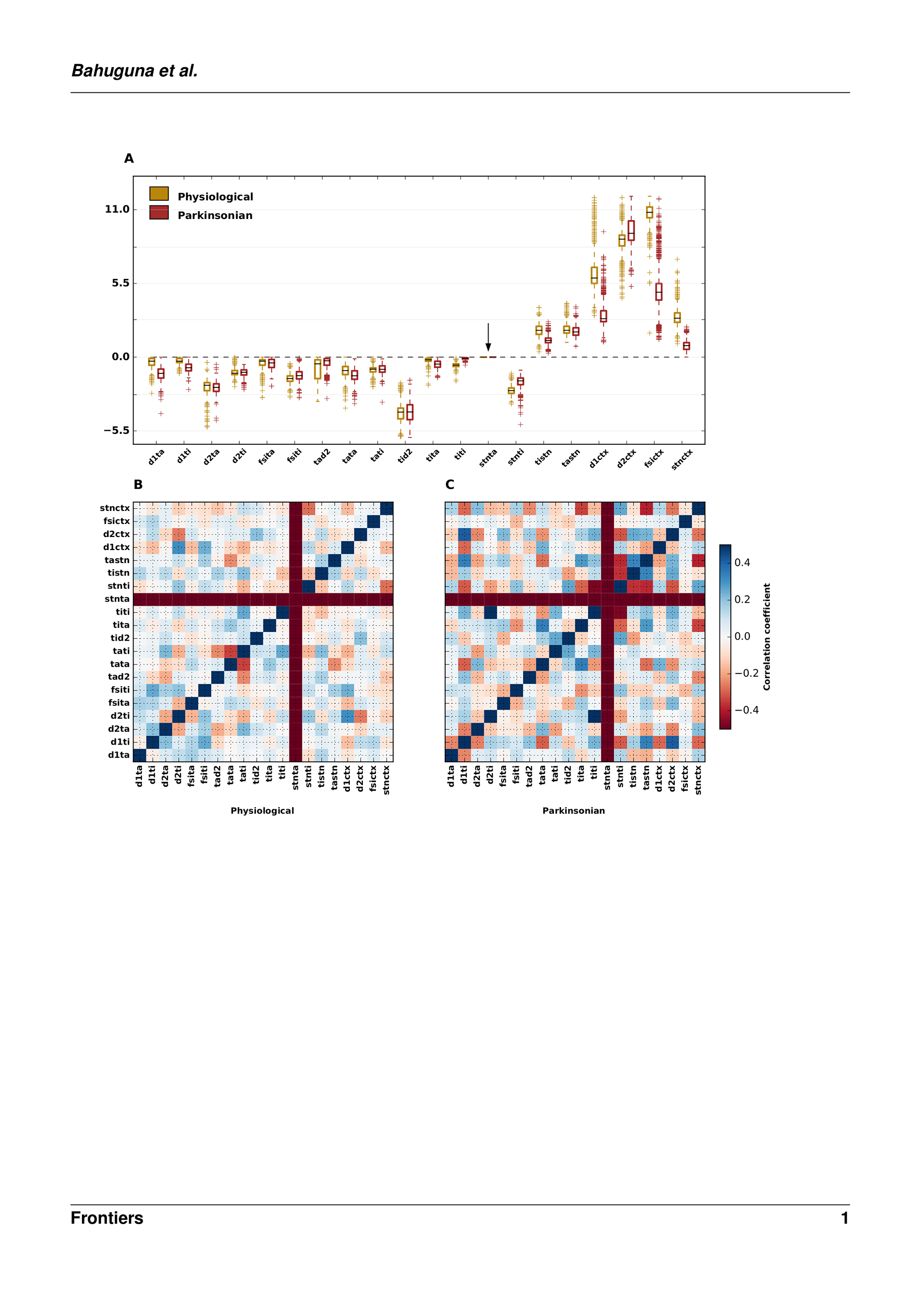

Supplement: Supplementary Figure 4 — Parameters distributions when JSTN,TA was fixed to zero. (A–C) same as Figures 4A–C. [file Image4.TIFF]

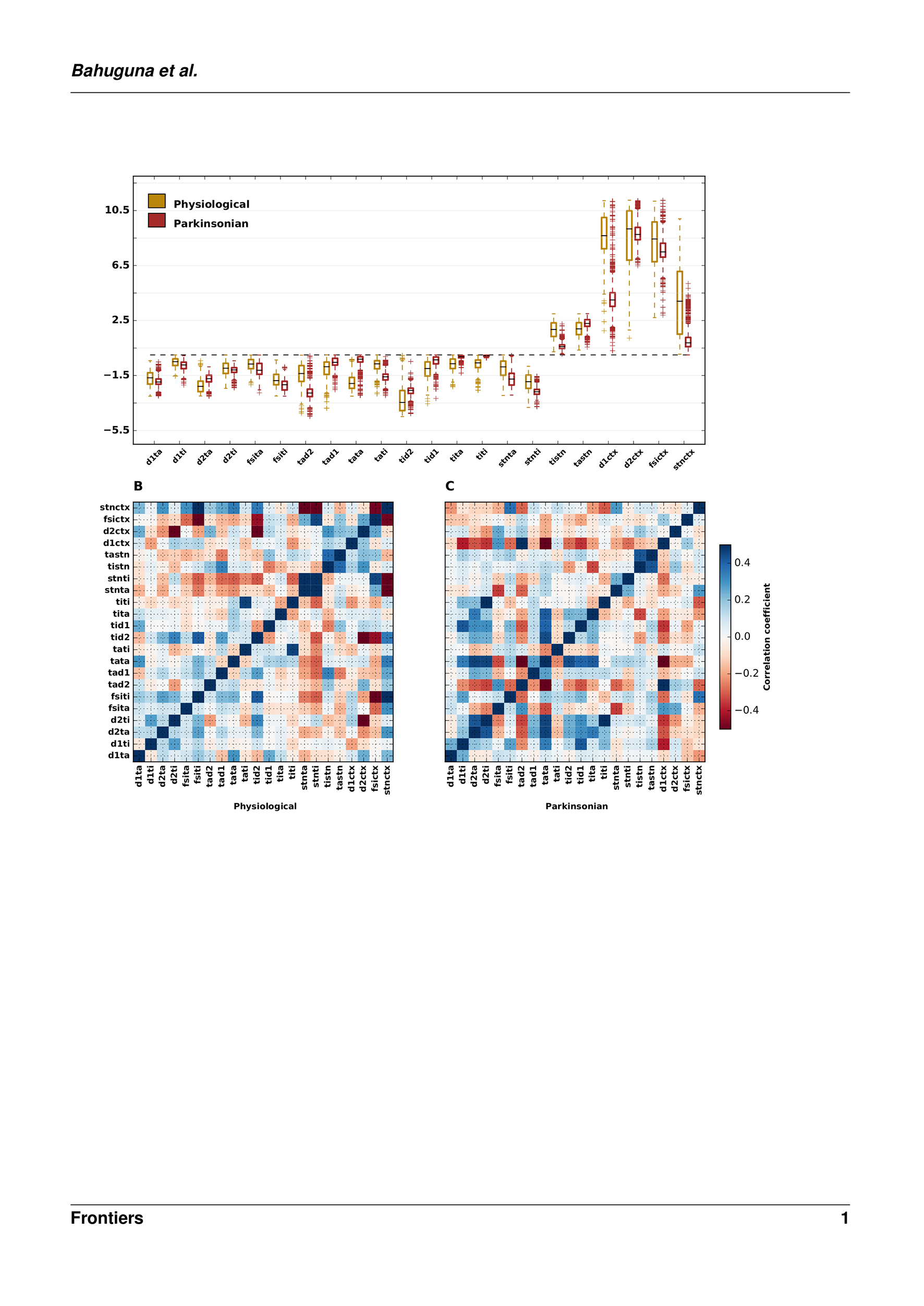

Supplement: Supplementary Figure 5 — Parameter distributions when connections from D1 to GPe-TA/TI are included as free parameters in the genetic algorithm. (A–C) same as Figures 4A–C. [file Image5.TIFF]

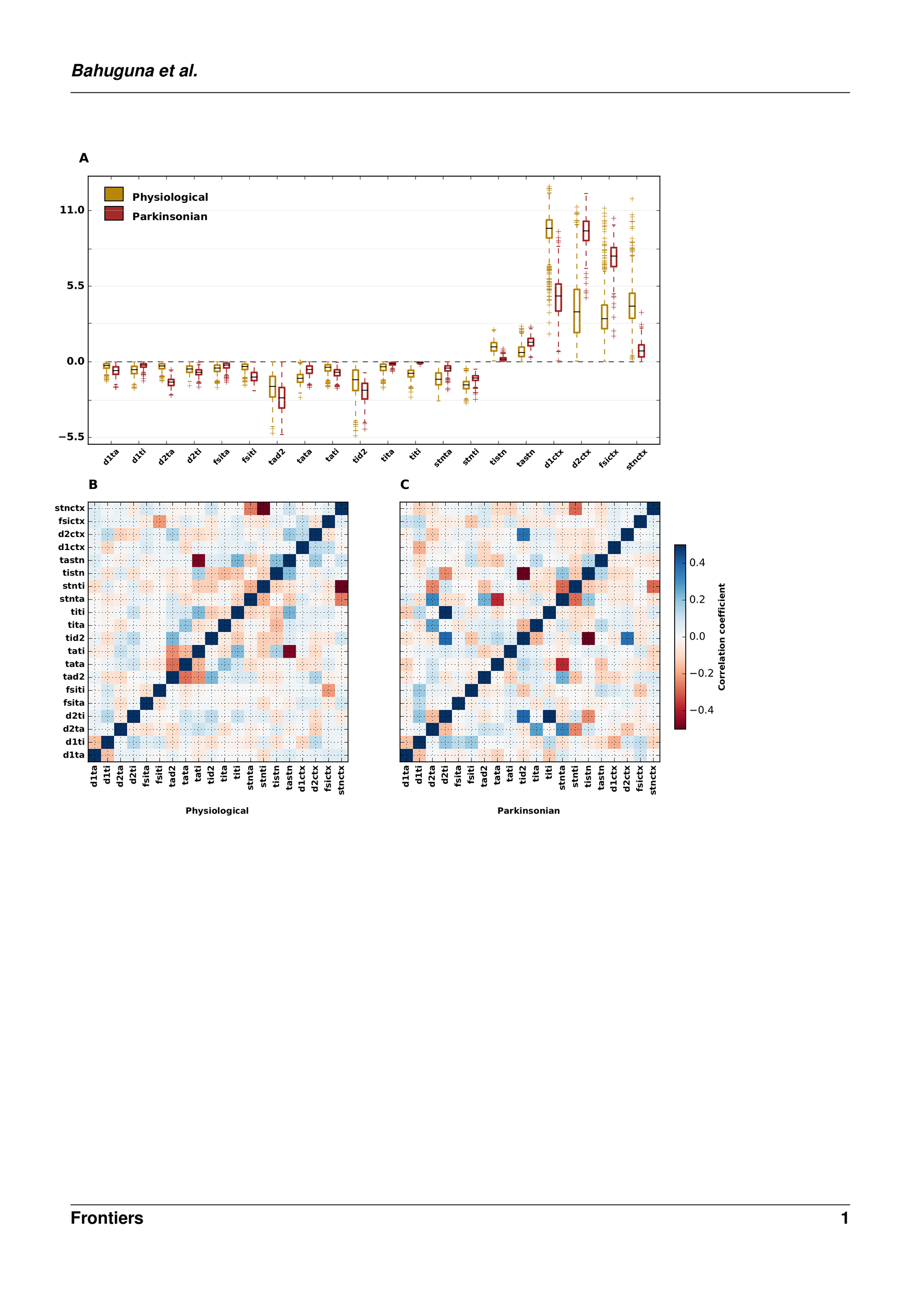

Supplement: Supplementary Figure 6 — Parameter distributions when GPi suppression (GS) and Susceptibility to oscillations (SO) are used as constraints. (A–C) same as Figures 4A–C. [file Image6.TIFF]

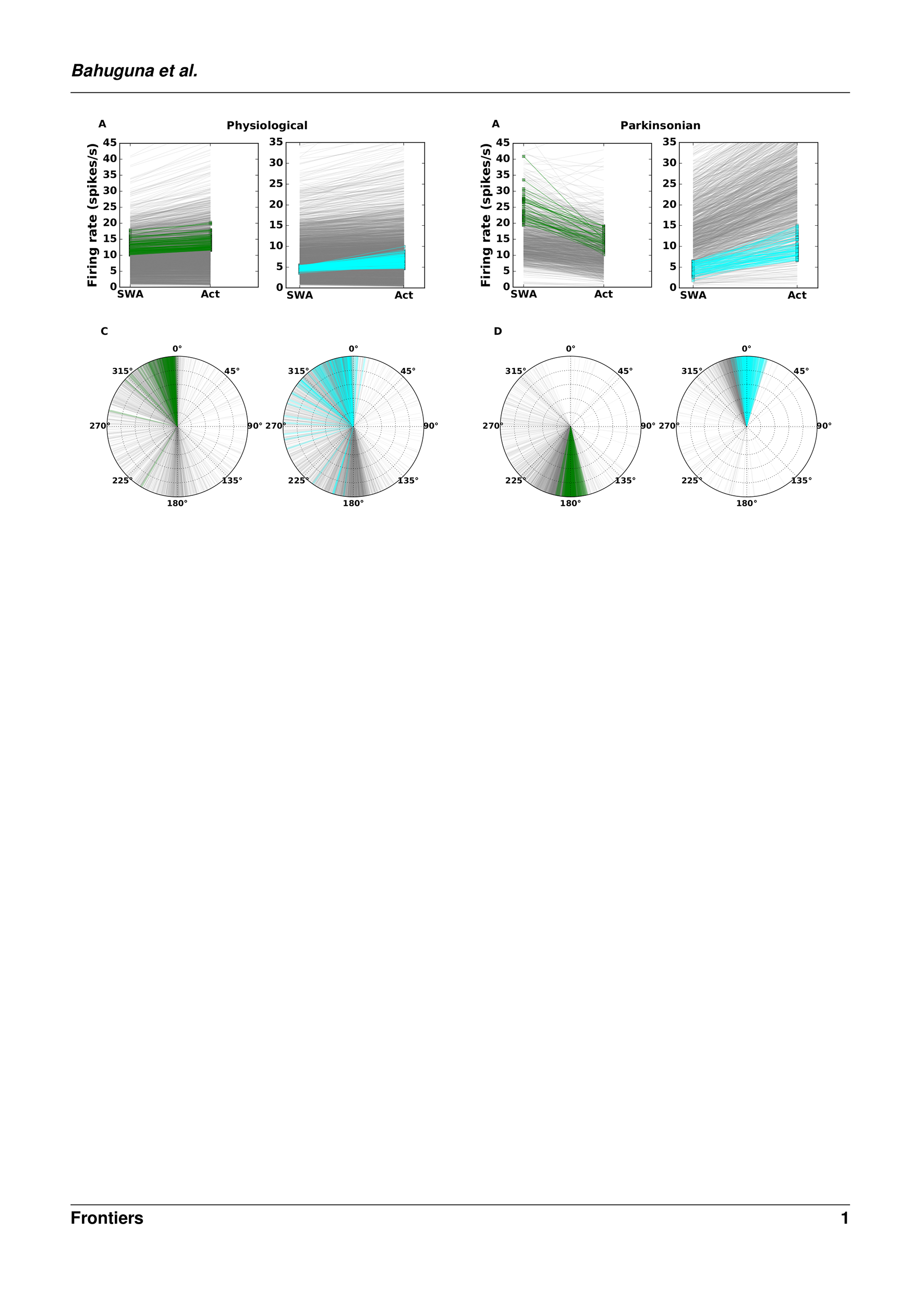

Supplement: Supplementary Figure 7 — Firing rates and phase relationships for valid and invalid networks when constrained using GS and SO. Gray lines: invalid networks. Cyan lines: Valid networks for GPe-TA. Green lines: Valid networks for GPe-TI. Compare with Figure 2. [file Image7.TIFF]
